# ACAD10 and ACAD11 allow entry of 4-hydroxy fatty acids into β-oxidation

**DOI:** 10.1007/s00018-024-05397-8

**Published:** 2024-08-22

**Authors:** Stéphanie Paquay, Julia Duraffourd, Marina Bury, Isaac P. Heremans, Francesco Caligiore, Isabelle Gerin, Vincent Stroobant, Jean Jacobs, Aymeric Pinon, Julie Graff, Didier Vertommen, Emile Van Schaftingen, Joseph P. Dewulf, Guido T. Bommer

**Affiliations:** 1 Metabolic Research Group, de Duve Institute & WELRI, Université Catholique de Louvain, 1200, Brussels, Belgium; 2 WELBIO Department, WEL Research Institute, avenue Pasteur, 6, 1300 Wavre, Belgium; 3 Department of Pediatric Neurology and Metabolic Diseases, Cliniques Universitaires St. Luc, Université Catholique de Louvain, 1200, Brussels, Belgium; 4 Ludwig Institute for Cancer Research, 1200 Brussels, Belgium; 5 Protein Phosphorylation Unit, de Duve Institute & MASSPROT Platform, Université Catholique de Louvain, 1200, Brussels, Belgium; 6 Department of Laboratory Medicine, Cliniques Universitaires St. Luc, Université Catholique de Louvain, 1200, Brussels, Belgium

**Keywords:** Beta-oxidation, 4-hydroxy fatty acids, ACAD10, ACAD11, Phosphohydroxyacyl-CoA, Haloacid dehalogenase domain

## Abstract

Hydroxylated fatty acids are important intermediates in lipid metabolism and signaling. Surprisingly, the metabolism of 4-hydroxy fatty acids remains largely unexplored. We found that both ACAD10 and ACAD11 unite two enzymatic activities to introduce these metabolites into mitochondrial and peroxisomal β-oxidation, respectively. First, they phosphorylate 4-hydroxyacyl-CoAs via a kinase domain, followed by an elimination of the phosphate to form enoyl-CoAs catalyzed by an acyl-CoA dehydrogenase (ACAD) domain. Studies in knockout cell lines revealed that ACAD10 preferentially metabolizes shorter chain 4-hydroxy fatty acids than ACAD11 (i.e. 6 carbons versus 10 carbons). Yet, recombinant proteins showed comparable activity on the corresponding 4-hydroxyacyl-CoAs. This suggests that the localization of ACAD10 and ACAD11 to mitochondria and peroxisomes, respectively, might influence their physiological substrate spectrum. Interestingly, we observed that ACAD10 is cleaved internally during its maturation generating a C-terminal part consisting of the ACAD domain, and an N-terminal part comprising the kinase domain and a haloacid dehalogenase (HAD) domain. HAD domains often exhibit phosphatase activity, but negligible activity was observed in the case of ACAD10. Yet, inactivation of a presumptive key residue in this domain significantly increased the kinase activity, suggesting that this domain might have acquired a regulatory function to prevent accumulation of the phospho-hydroxyacyl-CoA intermediate. Taken together, our work reveals that 4-hydroxy fatty acids enter mitochondrial and peroxisomal fatty acid β-oxidation via two enzymes with an overlapping substrate repertoire.

**Supplementary Information:**

The online version contains supplementary material available at 10.1007/s00018-024-05397-8.

## Introduction

Fatty acids with a hydroxyl group on carbon 3 are well-known intermediates of β-oxidation and fatty acid synthesis. In addition, different hydroxylated FAs can be generated when unsaturated fatty acids are oxidized either by dedicated enzymes or by exposure to reactive oxygen species [1]. The resulting hydroxylated compounds play diverse roles: some are signaling molecules [2, 3], some are degradation products of active epoxides [4], some are structural lipids [5, 6], and some are breakdown products of damaged lipids [7, 8]. Beyond this endogenous production, a variety of hydroxylated fatty acids can be taken up with the food or generated by intestinal microbiota [9–12].

**Fig. 1:**
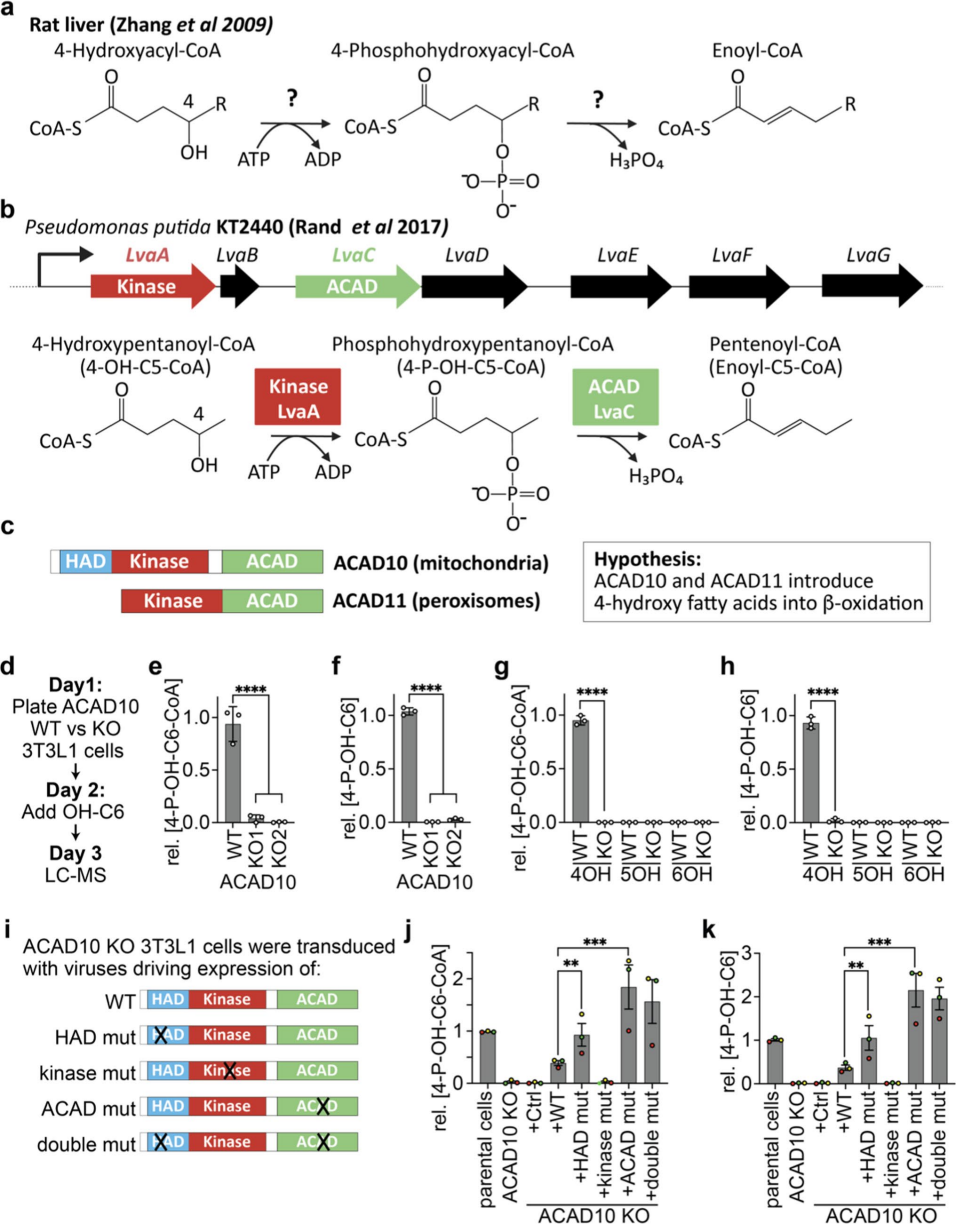
ACAD10 is required for the formation of 4-phosphohydroxyhexanoyl-CoA from 4-hydroxyhexanoic acid in 3T3L1 cells. **a** Schematic representation of the mammalian metabolic pathway of 4-hydroxy fatty acids [8, 15, 17]. **b** Schematic representation of the metabolism of 4-hydroxypentanoyl-CoA by proteins encoded by the Lva operon reported by Rand and colleagues [18], where the kinase LvaA leads to the formation of 4-phosphohydroxypentanoyl-CoA that is subsequently converted to pentenoyl-CoA by LvaC, a member of the acyl-CoA dehydrogenase (ACAD) family, followed by a partial conversion into 3-hydroxypentanoyl-CoA. **c** Schematic representation of domain organization of ACAD10 and ACAD11 proteins, where the kinase domains of ACAD10 and ACAD11 show 29% identity with LvaA from *Pseudomonas putida* KT2440, and the ACAD domains show 62% and 56% identity with LvaC, respectively. **d**–**f** Experimental setup (**d**), and LC–MS quantification of 4-phosphohydroxyhexanoyl-CoA (4-P-OH-C6-CoA) (**e**) and 4-phosphohydroxyhexanoate (4-P-OH-C6) (**f**) in parental 3T3L1 cells and two independent ACAD10 knockout clones after 16 h treatment with 2 mM racemic mixture of 4-hydroxyhexanoate (4-OH-C6). **g**, **h** Quantification of the corresponding 4-P-OH-C6-CoA (**g**) and 4-P-OH-C6 (**h**) in ACAD10 wild-type and knockout 3T3L1 cells after treatment for 16 h with 2 mM of 4-hydroxy-, 5-hydroxy- or 6-hydroxyhexanoate. **i** Schematic representation of the mutants used in panel **j** and **k**. **j**, **k** LC–MS quantification of 4-P-OH-C6-CoA (**j**) and 4-P-OH-C6 (**k**) in ACAD10 wild-type and knockout 3T3L1 cells that were transduced with lentiviral expression vectors for ACAD10 with the presumptively inactivating mutations in specific domains. ‘Ctrl’ denotes an empty vector. In all panels, signals were normalized to total ion current (TIC) and are presented relative to the median of the signal observed in parental cells treated with 4-OH C6 (in each experiment). Data are means ± SD of three independent experiments performed, each containing three independent samples. Identical colors in panel **j**, **k** are from the same experiment. Asterisks indicate significance in post-hoc testing after ANOVA

Outside of β-oxidation, mammalian cells can degrade hydroxylated fatty acids in Ω-oxidation (hydroxyl group on pre-/terminal carbon) and α-oxidation (hydroxyl group on carbon 2) [13, 14]. Curiously, little is known about how mammalian cells metabolize 4-hydroxy (4-OH) fatty acids except for the observation that their degradation involves the formation of a 4-phospho-hydroxy-acyl-CoA (4P-OH-acyl-CoA) intermediate [8, 15] (Fig. 1a). It has also been reported that this degradation pathway might be involved in the metabolism of 4-hydroxynonenal [16, 17], a reactive intermediate that forms when reactive oxygen species damage unsaturated fatty acids. Yet, up to now the enzymatic repertoire required for this metabolism has remained unknown.

The bacterial metabolism of levulinic acid (i.e. 4-ketopentanoic acid) depends on a series of enzymes that are located in the Lva operon of *Pseudomonas putida* KT2440 [18] (Fig. 1b)*.* This pathway involves the formation of a 4-phosphohydroxypentanoyl-CoA from 4-hydroxypentanoyl-CoA by the kinase LvaA, reminiscent of what had been reported to form during mammalian metabolism of 4-OH fatty acids. Subsequently, the 4-P-OH-acyl-CoA intermediate undergoes a phosphate elimination reaction catalyzed by the protein LvaC, which belongs to the family of acyl-CoA dehydrogenases. This leads to the production of pentenoyl-CoA that is partially hydrated by the same enzyme to form 3-OH-pentanoyl-CoA (not shown), a metabolite of fatty acid β-oxidation. These observations strongly suggest that both mammals and bacteria employ similar pathways to metabolize 4-OH fatty acids.

We report here that the kinase domain and the acyl-CoA dehydrogenase (ACAD) domain of human ACAD10 and ACAD11 catalyze both reactions required to introduce 4-OH-acyl-CoAs into β-oxidation and represent the missing players in the metabolism of 4-OH fatty acids. We also discovered that ACAD10 is cleaved between the kinase and the ACAD domain. Furthermore, we found that the activity of the kinase domain is restrained by the HAD domain, suggesting that the first step of 4-OH-acyl-CoA metabolism in mitochondria is regulated. This opens the door for future explorations aiming to understand how 4-OH fatty acid metabolism is integrated in mitochondrial metabolism.

## Results

### ACAD10 is required for the formation of 4-phosphohydroxyhexanoyl-CoA in 3T3L1 cells

We noted that the proteins ACAD10 and ACAD11 contain a kinase domain sharing 29% sequence identity with *Pseudomonas putida* KT2440 LvaA, and an ACAD domain sharing 62% and 56% sequence identity with LvaC, respectively (Fig. 1c, S1a-c). Furthermore, related kinases from several other bacterial species showed even higher similarity, reaching 51% and 38% identity to the kinase domains of ACAD10 and ACAD11, respectively (Fig. S1d). We therefore hypothesized that ACAD10 and ACAD11 might reunite the two activities required for the metabolism of 4-OH fatty acids within single polypeptide chains.

To test this hypothesis, we inactivated ACAD10 using CRISPR/Cas9 in the 3T3L1 preadipocyte cell line (Fig. S2) and measured metabolites by liquid chromatography coupled to mass spectrometry (LC–MS) after overnight treatment with 2 mM 4-hydroxyhexanoate (4-OH-C6) (Fig. 1d). Parental cells produced 4-phosphohydroxyhexanoyl-CoA (4-P-OH-C6-CoA) (Fig. 1e), which was almost completely abolished in two independent knockout clones. We also noted a concomitant production of 4-phosphohexanoate (4-P-OH-C6) only in parental cells, which is likely produced by hydrolysis of the CoA adduct (Fig. 1f). The formation of the P-OH-acyl-CoAs was specific for 4-OH fatty acids, since no formation was observed when cells were incubated with 5-hydroxy- or 6-hydroxyhexanoate (Fig. 1g, h). This clearly demonstrated that ACAD10 is required for the formation of the 4-P-OH-acyl-CoA intermediate.

To delineate the contribution of distinct ACAD10 domains, we restored ACAD10 in knockout cell lines using recombinant lentiviruses driving expression of either wild-type ACAD10 protein or variants carrying different mutations that presumptively inactivate specific domains of ACAD10 (Fig. 1i). Within the kinase domain, we replaced the conserved aspartate 463 with alanine, targeting a residue that is known to be required for the activity of related domains (Fig. S1b) [19]. Within the ACAD domain, we converted aspartate 1040 to alanine, given that a corresponding glutamate residue plays a key role in the catalytic cycle of other acyl-CoA dehydrogenases (Fig. S1c) [20]. We also converted aspartate 48 into alanine in the haloacid dehalogenase (HAD) domain that is present in the N-terminus of ACAD10. Many members of this family show phosphatase activity that depends on a DxD motif (Fig. S1a) [21]. Given that only the first of the invariant aspartate residues is present in ACAD10, we reasoned that this domain might somehow affect the metabolism of 4-hydroxyacyl-CoA, perhaps in a way that does not require a phosphatase activity.

Re-expression of wild-type ACAD10 led to the re-appearance of the 4-P-OH-C6-CoA and 4-P-OH-C6 peaks (Fig. 1j, k), confirming that ACAD10 is indeed required for their formation. Yet, levels only reached approximately 50% of what we observed in parental cells, suggesting that ACAD10 levels in the rescue cell lines remained below levels observed in parental cells. Consistent with this, ACAD10 expression levels in these cell lines remained below the detection limit in a western blot analysis (data not shown). Formation of 4-P-OH-C6-CoA remained undetectable when we expressed ACAD10 with a mutation in the kinase domain, indicating that this domain is required for its formation. In contrast, compared to wild-type ACAD10, the protein carrying a mutation in the ACAD domain led to fourfold higher 4-P-OH-C6-CoA levels, in line with our hypothesis that this domain is required to metabolize this phosphorylated metabolite. Curiously, both 4-P-OH-C6-CoA and 4-P-OH-C6 were approximately twofold higher in knockout cell lines rescued with HAD-mutant ACAD10 in comparison to cell lines rescued with the wild-type protein (Fig. 1j, k). This indicated that the HAD domain somehow affects production or utilization of 4-P-OH-C6-CoA (see also results presented later in Fig. 4). Regardless, our findings demonstrated that ACAD10 is required for the formation and utilization of 4-P-OH-acyl-CoAs known to be involved in the metabolism of 4-OH fatty acids.

### ACAD10 is cleaved into two distinct proteins, likely via the mitochondrial processing peptidase

**Fig. 2:**
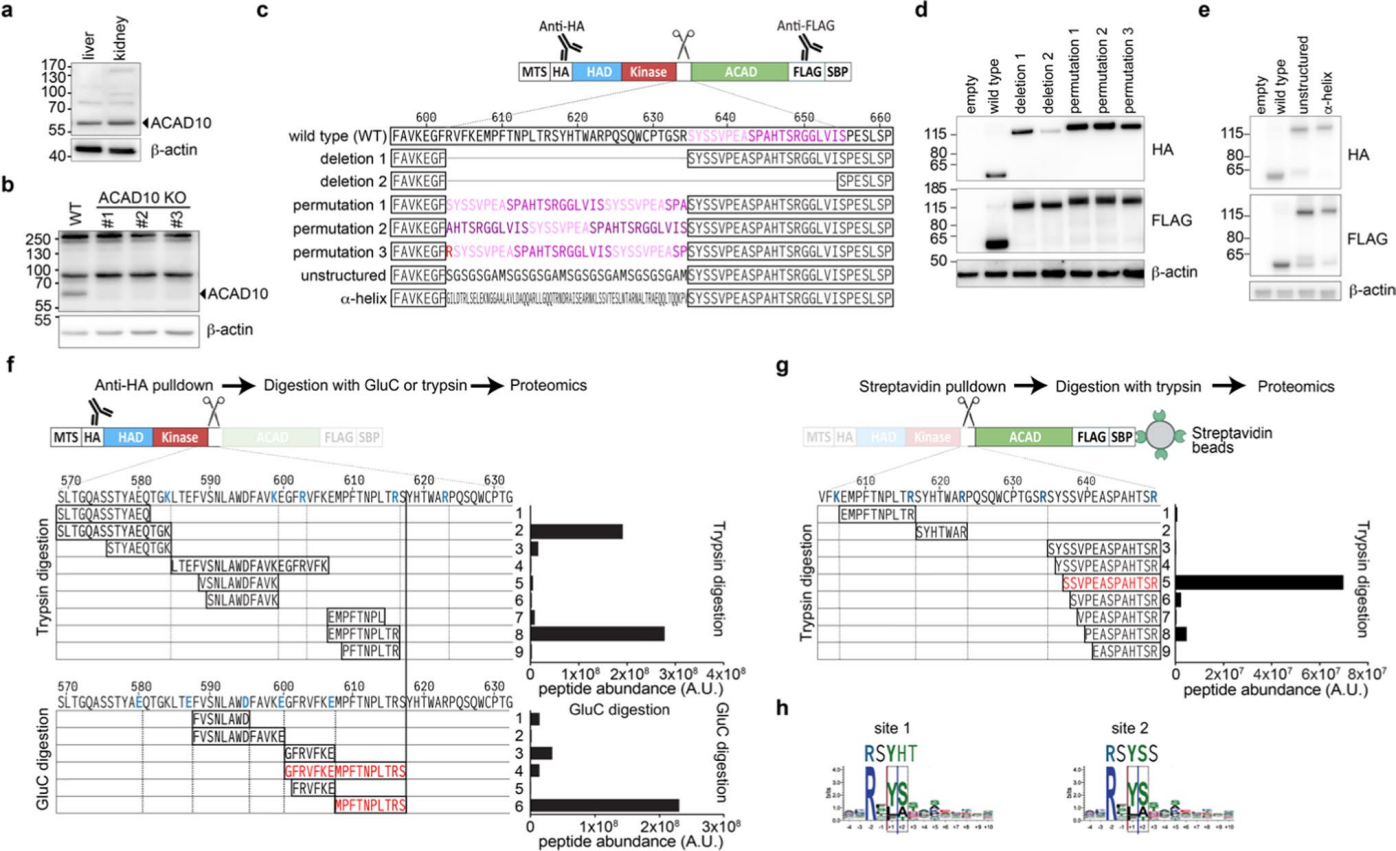
ACAD10 undergoes internal cleavage in consensus sequences for the mitochondrial processing peptidase to form two independent proteins. **a**, **b** Western blot analysis of mouse liver and kidney lysates (**a**) and parental HepG2 wild-type cells or two independent ACAD10 knockout clones (**b**) using the indicated antibodies. Note that a band at approximately 60 kDa in wild-type cells is lost in knockout cells, whereas other high molecular weight bands are unaffected. **c** Schematic representation of the expression constructs utilized in panels **d** and **e**. ‘HA’ and ‘FLAG’ denote epitope tags. MTS represents the mouse Cox8 mitochondrial targeting sequence. SBP stands for streptavidin-binding peptide. Note that an α-helix presenting approximately the same length as an extended unstructured loop was chosen. To achieve this, a longer amino acid sequence was required. **d**, **e** Western Blot analysis of ACAD10-deficient HepG2 cell lines upon re-expression of the indicated constructs. The anti-HA antibody recognizes the N-terminal part and the anti-FLAG antibody recognizes the C-terminal part of the protein. **f**, **g** Proteomic analysis to determine the precise cleavage sites of ACAD10. Anti-HA antibody (**f**) or Streptavidin beads (**g**) were used to pulldown the N-terminal and C-terminal part of ACAD10 from lysates of cell lines expressing wild-type ACAD10 with N-terminal HA and C-terminal FLAG-SBP tags. Peptides observed upon cleavage with GluC or trypsin are indicated on the left, and their abundance is indicated on the right. The blue color is used to highlight expected cleavage sites during sample processing, whereas the red color highlights abundant peptides that can only be the result of a cleavage in cells prior to sample processing. **h** The cleavage sites correspond to the consensus motif for the cleavage by the mitochondrial processing peptidase system [29]. A.U. = arbitrary units

ACAD10 is a mitochondrial protein [22, 23]. As such, we expected that an N-terminal mitochondrial targeting sequence (MTS) would be cleaved off during mitochondrial import [24]. When we analyzed mouse tissue samples or different cell lines using an antibody directed against the N-terminus of ACAD10, we barely detected a band at the predicted molecular weight of 130 kDa (Fig. 2a, b), even if we accounted for the removal of a MTS. In contrast, we detected a strong band with an apparent molecular weight of ± 60 kDa, which was absent when we inactivated ACAD10 in the HepG2 hepatoblastoma cell line (Fig. 2b, Fig. S3). This indicated that ACAD10 was cleaved during its maturation. Based on the apparent molecular weight of the N-terminal fragment, a cleavage between the kinase and the ACAD domains seemed most likely.

To understand where ACAD10 might be cleaved, we used the Attention DisOrder PredicTor (ADOPT) pipeline to predict unstructured regions that might be accessible to proteases [25]. This revealed a putative unstructured region between amino acids 606 and 653 (Fig. S4a, highlighted in cyan). The precise structure of ACAD10 is unknown, but an Alphafold structure is available (Fig. S4b). In this model, the three individual domains of ACAD10 are predicted with high confidence, but no structural prediction at all was available for the segment linking the kinase to the ACAD domain. We therefore wanted to investigate whether this linker domain was required for ACAD10 cleavage. First, we generated an experimental system that allowed us to detect both the N- and the C-terminal part of ACAD10. To this end, we added a FLAG tag and a streptavidin-binding peptide to the C-terminus and replaced the endogenous presumptive MTS of ACAD10 with the MTS of mouse COX8A and an HA tag (Fig. 2c). In cells expressing wild-type ACAD10, the anti-FLAG antibody mainly detected a band with an apparent molecular weight corresponding to the size of the ACAD domain, while the anti-HA signal was found at the size corresponding to the combination of the HAD and the kinase domain (Fig. 2d, e). The predicted full-length band remained barely detectable. In contrast, a partial or complete deletion of the predicted unstructured loop (Fig. 2c, deletion 1 and 2) completely prevented cleavage of ACAD10 (Fig. 2d, lanes 3 and 4). We reasoned that this lack of cleavage could be due to the absence of the recognition site required for cleavage, or due to shortening of the unstructured loop. To test this, we replaced the first part of the unstructured loop with permutations of the second part while maintaining the overall length of this area (Fig. 2c, permutation 1–3). Again, we did not see any production of the cleaved ACAD10 protein (Fig. 2d, lanes 5–7). We also mainly detected the full length uncleaved protein when we overexpressed ACAD10 protein where the predicted unstructured loop was replaced by an unrelated unstructured sequence or an α-helix of comparable length derived from the *E. coli* protein colicin [26] (Fig. 2e). Overall, these data suggested that ACAD10 is cleaved in the unstructured region linking the kinase and the ACAD domain in a manner that depended on specific sequences in this area.

To precisely locate the cleavage site, we purified ACAD10 by affinity purification via the N-terminal HA-tag or the C-terminal streptavidin-binding peptide, followed by proteomic analysis. Peptides were digested either with GluC (cleaving after glutamate or aspartate residues) or with trypsin (cleaving after arginine and lysine) giving rise to an overlapping set of peptides. We reasoned that any cleavage occurring in cells should yield peptides that end at amino acids that do not correspond to GluC or trypsin cleavage sites. Furthermore, peptides beyond the cleavage sites should be of much lower abundance or absent. When we purified the N-terminal part, we observed that peptides beyond amino acid 617 were essentially absent (Fig. 2f). Furthermore, we found a very strong signal in the GluC digestion for a peptide that terminated with serine 617, which must have been produced by a proteolytic cleavage in cells (Fig. 2f, highlighted in red). Next, we analyzed ACAD10 purified via the C-terminal streptavidin-binding peptide. One of the most abundant peptides started with serine 637 (Fig. 2g, highlighted in red). Again, this peptide cannot be the result of a cleavage by trypsin since it is preceded by a tyrosine residue (rather than an arginine or lysine required for trypsin). Both experiments clearly delineated the cleavage site of ACAD10. Surprisingly, they did not converge on the same site, but rather suggested that two separate cleavage events occur in the process of the formation of mature ACAD10. Curiously, both cleavage sites perfectly correspond to the consensus sequence of the site that is recognized by the mitochondrial processing peptidase (MPP, Fig. 2h) and ancillary proteases, which usually cleave off N-terminal mitochondrial targeting sequences [27]. Consistent with this, prediction algorithms for such recognition sites predicted both cleavage sites when we ‘coerced’ them to predict internal cleavage sites [28, 29] (Fig. S4c and d). Likewise, they correctly identified the cleavage site of the normal mitochondrial targeting sequence of ACAD10, which we identified when we purified an N-terminal ACAD10 fragment in further experiments (Fig. S4e).

Mitochondrial processing peptidase (MPP) contains a pocket recognizing an arginine residue two residues before the cleavage site [30], explaining the importance of arginine residues in substrate recognition. To investigate the requirement of the predicted MPP cleavage sites, we generated expression constructs where arginines 616 and 634, next to the observed cleavage sites, were converted to alanine. We also targeted arginine 603 that also corresponded to a predicted MPP cleavage site. Single arginine mutants behaved identical to the wild type construct in western blot analysis with antibodies recognizing the C- and the N-terminus of ACAD10 via epitope tags (Fig. S5a-d), demonstrating that they were still efficiently cleaved. To explore the possibility of several concurrent or consecutive cleavage events, we mutated all three arginine residues (‘triple mutant’) and even a fourth residue, arginine 623, outside of a MPP consensus motif (‘quadruple mutant’). The intensity of the uncleaved protein in western blot was strongly increased in the triple and quadruple mutant (Fig. S5b). In parallel, the mature cleaved N-terminal and C-terminal parts were reduced by 50% (Fig. S5c-d). This indicated that the MPP consensus sites are required for efficient internal cleavage of ACAD10, similar to what had recently been reported for some yeast proteins [31, 32]. Yet, we were surprised that the triple mutant was still cleaved. To explore where cleavage occurred, we analyzed the C-terminal part produced from different mutants using a proteomic approach. For both the triple and quadruple mutants, we observed an increase in peptides N-terminal to the cleavage site of ACAD10 (Fig. S5e). Surprisingly, in both of these mutants the most abundant peptide remained the peptide starting with serine 637, opening the possibility that other proteases might contribute to ACAD10 cleavage.

Taken together, we conclude that ACAD10 is efficiently cleaved in two parts. Future studies will need to resolve whether proteases other than MPP contribute, or whether MPP does not absolutely require an arginine residue for cleavage of ACAD10.

### The activity of ACAD10 can be recapitulated by recombinant proteins

**Fig. 3:**
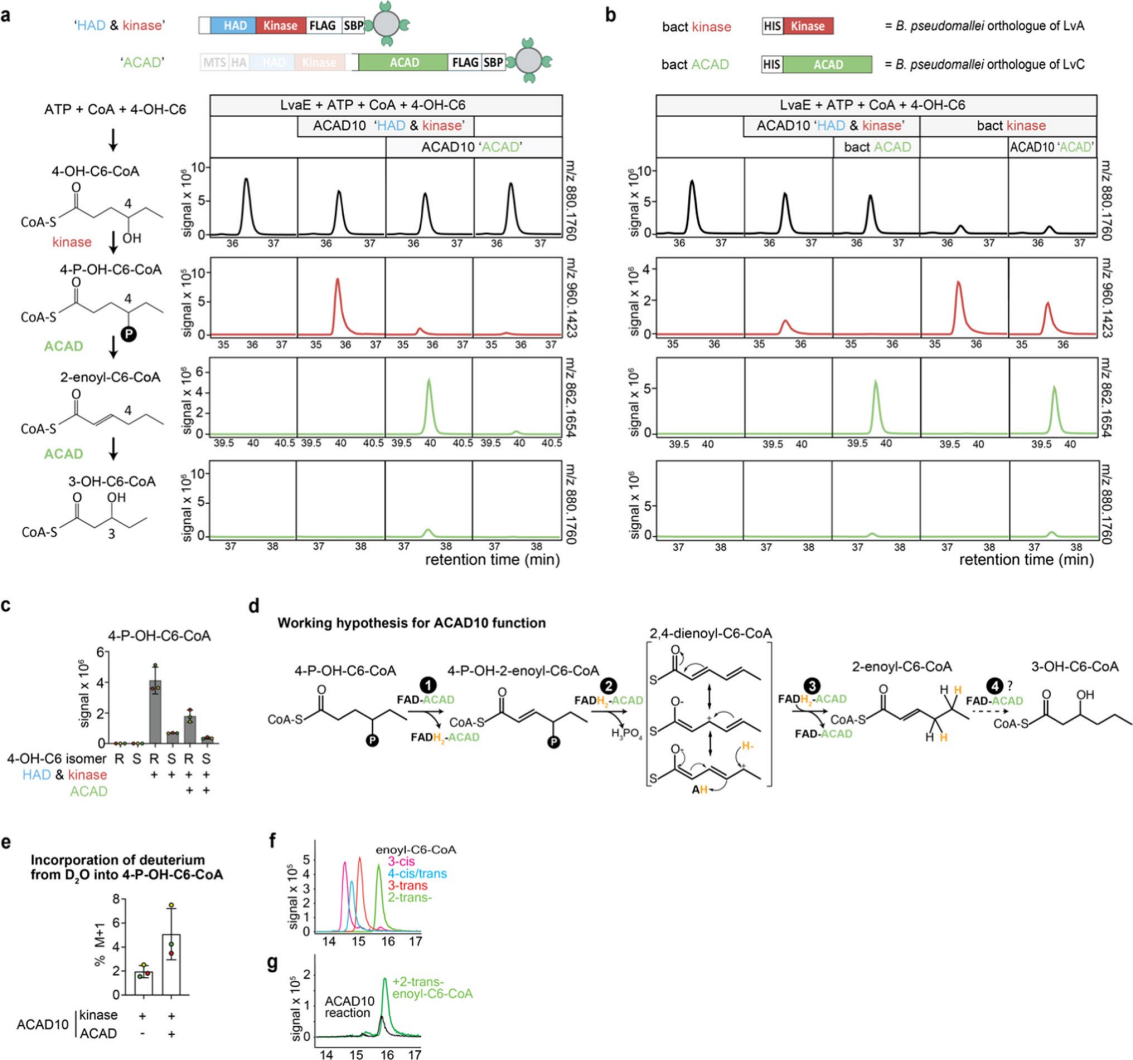
ACAD10 converts 4-hydroxyhexanoyl-CoA into 2-*trans*-hexenoyl-CoA and 3-hydroxyhexanoyl-CoA, similar to the bacterial orthologs LvA and LvaC. **a**, **b** Extracted ion chromatograms (EICs) for the expected m/z [M-H]^−^ of 4-hydroxyhexanoyl-CoA (4-OH-C6-CoA), 4-phosphohydroxyhexanoyl-CoA (4-P-OH-C6-CoA), hexenoyl-CoA or 3-hydroxyhexanoyl-CoA (3-OH-C6-CoA) obtained by LC–MS analysis of reactions in the presence and absence of the kinase and the ACAD domain of ACAD10 (**a**), or in reactions where the mammalian enzymes were replaced by the corresponding bacterial ortholog from *Burkholderia pseudomallei* (**b**). In both instances, 4-OH-C6-CoA was produced in situ using recombinant LvaE from ATP, Coenzyme A and 4-hydroxyhexanoate (4-OH-C6). **c** Activity of the N-terminal and C-terminal parts of ACAD10 were assessed on R- or S-4-OH-C6-CoA, demonstrating a strong preference of the kinase for the R- form. **d** Schematic representation of the catalytic cycle of ACAD10. **e** The relative abundance of the M + 1 form of R-4-P-OH-C6-CoA was assessed in the presence and absence of the ACAD domain of ACAD10 in a reaction where this metabolite was produced by the kinase domain of ACAD10 in the presence of 50% D_2_O. **f**, **g** Synthetic standards (**f**) and reaction products of ACAD10 (**g**) were analyzed by LC–MS. Spike-in indicates the addition of 2-*trans*-enoyl-C6-CoA into an ACAD10 reaction with R-4-OH-C6-CoA, demonstrating that ACAD10 produces a canonical metabolite of β-oxidation

To explore the enzymatic function of ACAD10, we overexpressed recombinant proteins in HEK293 cells and purified them via a C-terminal streptavidin-binding peptide. To account for the cleavage of ACAD10, the N-terminal part comprised amino acids 1 to 608 until briefly before the first internal cleavage site (Fig. S5). In contrast, the C-terminal part was purified using the full length ACAD10 protein (Fig. S5). Next, we incubated these proteins in a reaction where 4-OH-C6-CoA was produced in situ by the bacterial enzyme LvaE (Fig. 3a, column 1). Addition of the N-terminal part of ACAD10 led to the production of 4-P-OH-C6-CoA (Fig. 3a, column 2). This metabolite was consumed upon addition of the C-terminal part of ACAD10, which led to the appearance of hexenoyl-CoA and, to a lower extent, of 3-OH-C6-CoA (Fig. 3a, column 3, Fig. S5c), similar to what had been reported for the bacterial proteins LvaA and LvaC [18].

Next, we explored whether the corresponding bacterial proteins could indeed replace the function of ACAD10 domains. To this end, we replaced either the C-terminal part or the N-terminal part of ACAD10 with bacterial proteins related to the kinase and the ACAD domain, respectively. We chose to do this with proteins from *Burkholderia pseudomallei*, which showed even higher sequence homology to the ACAD10 domains in comparison to the previously analyzed proteins from *Pseudomonas putida*. In both cases, we observed that the bacterial ortholog indeed can replace the corresponding ACAD10 domain, corroborating that they are performing the same function (Fig. 3b).

4-OH fatty acids are chiral molecules that exist in either the R or S configuration. We therefore tested whether ACAD10 was specific to one or the other isomer. Incubation of the N-terminal part of ACAD10 (containing the kinase domain) in the presence of R-4-OH-C6-CoA led to approximately fourfold higher levels of 4-P-OH-C6-CoA than in the presence of S-4-OH-C6-CoA. While this revealed a preference of ACAD10 for the R configuration, an activity on the S form was still detectable both for the kinase and for the ACAD domain (Fig. 3c). A similar preference for the R configuration was also observed in cell lines supplemented either with R- or S-4-OH-C6 (data not shown). To which extent this substrate specificity has been acquired to act on specific metabolites in mammals, or whether this is the consequence of the substrate specificity of ancestral proteins is difficult to determine.

The kinase domain of ACAD10 produces 4-P-OH-acyl-CoAs. Subsequently, the ACAD domain forms hexenoyl-CoAs that are, in part, hydrated to 3-hydroxy-acyl-CoAs (Fig. 3d). ACADs usually catalyse the formation of a carbon–carbon double bond in acyl-CoAs. This proceeds via the removal of a proton from carbon 2 and the transfer of a hydride ion to FAD. When we incubated 4-P-OH-C6-CoA in the presence of deuterated water, the addition of the C-terminal part of ACAD10 led to an incorporation of 1 deuterium atom into the substrate. This indicated that the ACAD domain elicited a reversible proton transfer onto the substrate, consistent with the catalytic mechanism of other ACADs (Fig. 3e).

Next, we explored the location of the double bond in the enoyl-CoA formed by ACAD10. When comparing the products of ACAD10 with synthetic standards, we observed coelution with 2-*trans*-hexenoyl-CoA (Fig. 3f, g), a metabolite of β-oxidation. This indicated that, similar to other ACADs, the ACAD domain of ACAD10 introduces a double bond between carbon 2 and carbon 3.

ACAD10 and ACAD11 act on 4-P-OH-acyl-CoAs, which are bulkier and more charged than acyl-CoAs. Consistent with this, ACAD10 and ACAD11 contain a motif with a highly conserved proline residue (Pro759 and Pro463 for human ACAD10 and ACAD11, respectively, in Fig. S7), that is not shared with other ACADs. Based on a crystal structure for ACAD11 (PDB 2wbi) and the Alphafold model for ACAD10 [33], these proline residues disrupt an α-helix found in the corresponding region of other ACADs, creating an expanded catalytic site cavity. Furthermore, both enzymes contain a conserved arginine residue (Arg 913 in human ACAD10 and Arg 627 in human ACAD11) that might help bind to the phosphate group.

In *bona fide* acyl-CoA dehydrogenases, electrons from the substrate are transferred to an enzyme-bound FAD to form FADH_2_, which is re-oxidized by transfer of the electrons to a secondary electron acceptor such as the electron-transfer flavoprotein (ETF) [34]. Curiously, the ACAD10 reaction did not require a terminal electron acceptor, indicating that the electrons from the substrate are somehow donated back to the substrate. Of note, the presence of a double bond next to a phosphate group facilitates the phosphate elimination in reactions catalyzed by lyases of the EC 4.2.3 group. Thus, the most likely reaction path would be the transient formation of a 2,4-dienoyl-CoA with a conjugated double bond system that is reduced back to 2-*trans*-enoyl-CoA.

Overall, our experiments with recombinant ACAD10 demonstrated that ACAD10 metabolizes 4-OH-C6-CoA through a similar pathway as members of the Lva operon to form 2-*trans*-enoyl-CoA [18]. After phosphorylation by the kinase domain, the substrate likely undergoes sequential oxidation, phosphate elimination and reduction reactions catalyzed by the ACAD domain (Fig. 3d).

### The HAD domain restrains the activity of the kinase domain

**Fig. 4:**
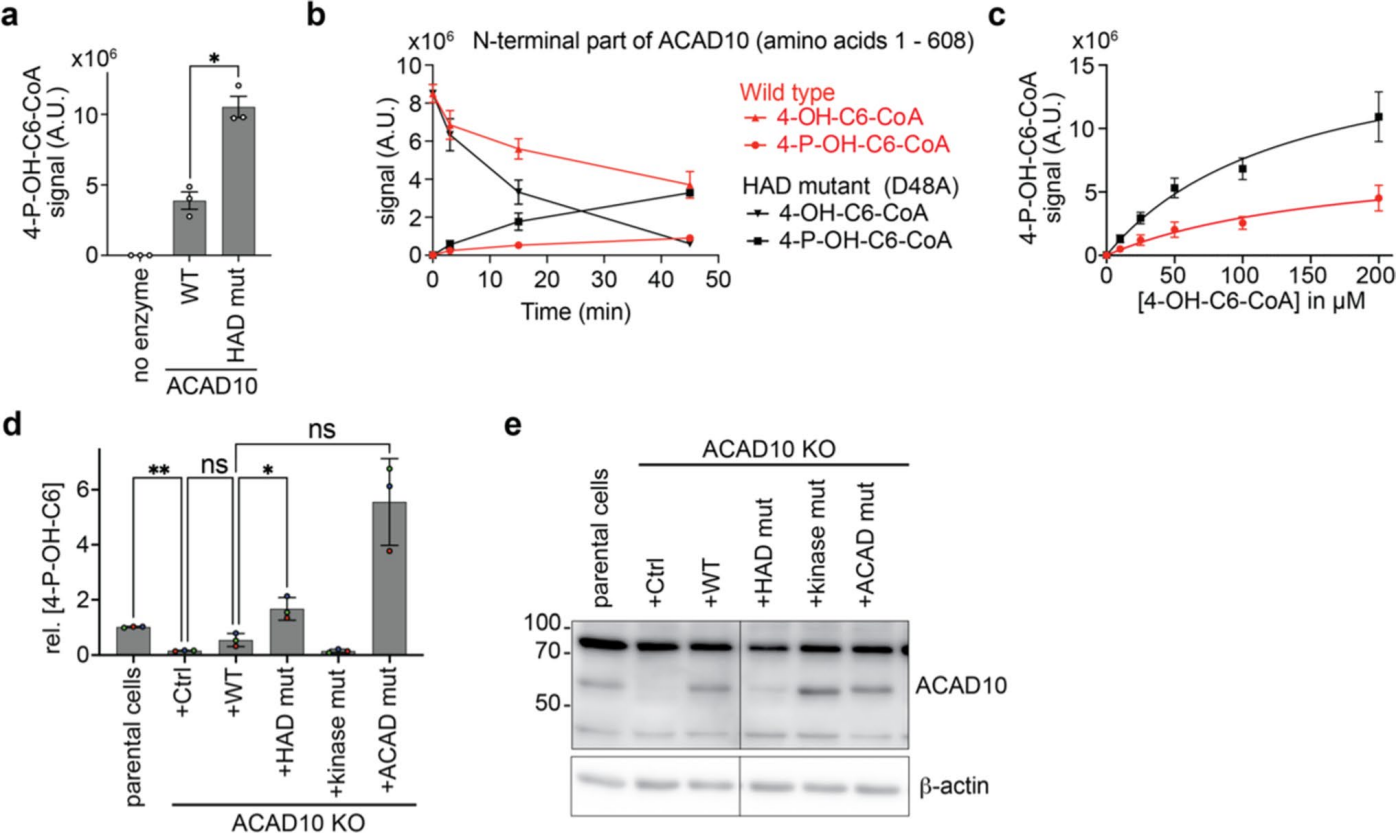
The HAD domain restrains the activity of the kinase domain both in vitro and in cells. **a**–**c** Production of 4-phosphohydroxyhexanoyl-CoA (4-P-OH-C6-CoA) from purified R-4-hydroxyhexanoyl-CoA (R-4-OH-C6-CoA) was assessed using LC–MS in reactions with the N-terminal part of wild-type ACAD10 or proteins with a mutation in a conserved residue of the HAD domain. Data were obtained after 60 min with 25 nM enzyme and 50 µM substrate (**a**), at different times with 25 µM substrate and 3.5 nM enzyme (**b**) or at the indicated substrate concentrations for 60 min with 25 nM enzyme (**c**). The raw signal is presented in arbitrary units (A.U.). **d** 4-P-OH-C6 levels after incubation with 2 mM of racemic mixtures of 4-OH-C6 for 16 h were assessed by LC–MS in samples from parental HepG2 cells or an ACAD10 knockout clone where ACAD10 was reconstituted with the mutations described in Fig. 1i. **e** Western blot analysis for the cell lines investigated in panel d. Metabolite quantifications are normalized to TIC and are presented relative to the median signal in parental cells within each experiment, as means ± SD of three independent experiments, consisting of three independent samples. Asterisks indicate significance in post hoc testing after ANOVA

After the cleavage of ACAD10, the kinase domain remains attached to the HAD domain. This opened the possibility that there might be a functional interaction between these two domains. We therefore explored the activity of the ACAD10 kinase domain in recombinant proteins with a wild-type HAD domain or with an HAD domain containing the mutation D48A targeting the remaining aspartate residue within the consensus site of HAD domains [21]. Incubation of this HAD-mutant protein with substrate consistently led to a significantly increased production of 4-P-OH-C6-CoA (Fig. 4a) compared to the wild-type protein. This was even more apparent when we followed this reaction over time (Fig. 4b) or when we assessed activity in the presence of different substrate concentrations (Fig. 4c). The most parsimonious explanation for this observation would be that the HAD domain simply dephosphorylates part of the 4-P-OH-C6-CoA produced by the kinase domain. Yet, consistent with the lack of a complete DxD motif characteristic for HAD phosphatases, we only observed negligible phosphatase activity on 4-P-OH-C6-CoA (data not shown) which could not explain the much lower production of 4-P-OH-C6-CoA by the wild-type enzyme in comparison to the HAD mutant protein.

Looking back to the results obtained in 3T3L1 cells, we had observed that the re-expression of HAD-mutant ACAD10 led to twofold higher levels of 4-P-OH-C6-CoA compared to the wild-type protein. Yet, given the low levels of ACAD10 expression in these experiments, we were not able to verify whether these observed differences might be caused by differences in protein expression. We therefore performed a similar experiment in HepG2 ACAD10 knockout cells. Loss of ACAD10 strongly reduced 4-P-OH-C6 levels which were partially restored upon re-expression of the wild-type protein. Again, HAD-deficient ACAD10 led to considerably higher 4-P-OH-C6 levels than the wild-type protein (Fig. 4d). In this case, expression levels of the HAD-deficient protein were lower than those of the wild-type protein (Fig. 4e), indicating that a difference in protein activity rather than a difference in protein abundance is causing the increased levels of 4-P-OH-C6 in the presence of HAD-deficient ACAD10. Taking together the data obtained with recombinant proteins and in cells, we conclude that the HAD domain somehow restrains the activity of the kinase domain. Whether this allows for an allosteric regulation of this enzyme by hitherto unknown metabolites will need to be explored in future studies.

### ACAD10 serves to preferentially metabolize shorter 4-hydroxy fatty acids than ACAD11

**Fig. 5:**
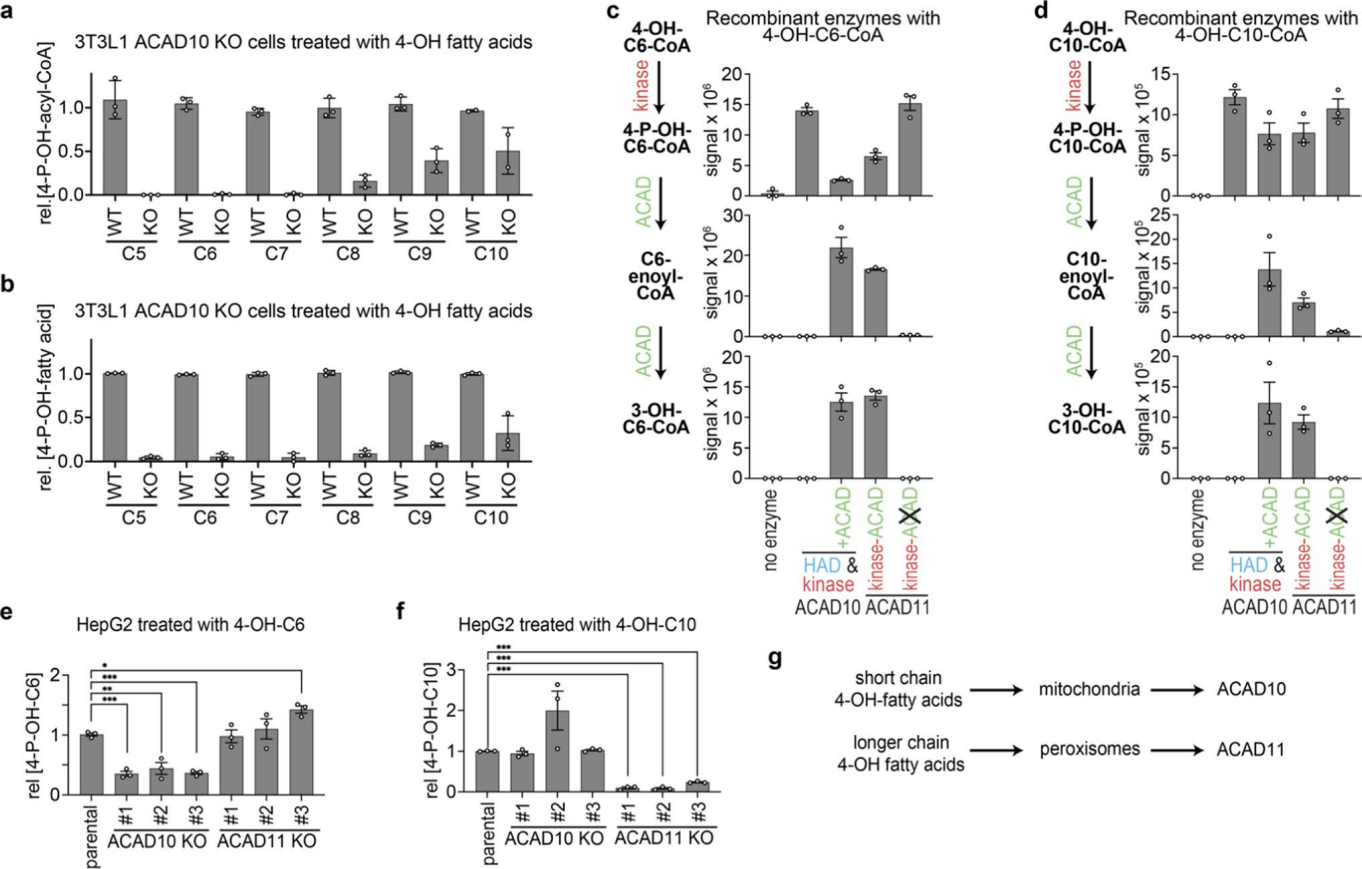
ACAD10 and ACAD11 serve to preferentially metabolize short- and long- chain 4-hydroxy fatty acids, respectively. **a**, **b** Quantification of 4-phosphohydroxyacyl-CoAs (4-P-OH-acyl-CoAs) (**a**) and 4-phosphohydroxy fatty acids (4-P-OH-fatty acids) (**b**) by LC–MS in parental and ACAD10 knockout 3T3L1 cells treated with 2 mM of racemic mixtures of 4-OH fatty acids with the indicated chain lengths for 16 h. Metabolite quantifications are normalized to TIC and are represented relative to median of the values obtained in parental cells within each experiment and within each treatment condition (means ± SD of three independent experiments, consisting of three independent samples). Asterisks indicate significance in post hoc testing after ANOVA. **c**, **d** Production of 4-P-OH-C6-CoA, 2-C6-enoyl-CoA and 3-OH-C6-CoA from 4-OH-C6-CoA (**c**) and production of 4-P-OH-C10-CoA, 2-C10-enoyl-CoA and 3-OH-C10-CoA from 4-OH-C10-CoA (**d**) was assessed by LC–MS reactions involving the ACAD10 N-terminal (‘kinase’) and C-terminal (‘ACAD’) part, as well as wild-type ACAD11 or ACAD11 with a mutation in the ACAD domain. Data are raw signals in arbitrary units. CoA derivatives of racemic mixtures of 4-OH C6 or 4-OH C10 were synthesized with LvaE or FadD, respectively, in the presence of 2.75 mM 4-OH fatty acid, 2.75 mM ATP, and 0.55 mM CoA, and incubated with 25 nM ACAD10 or ACAD11 for 60 min. **e**, **f** 4-P-OH-C6 (**e**) and 4-P-OH-C10 (**f**) were assessed in parental HepG2 cell lines where ACAD10 or ACAD11 had been genetically inactivated, after treatment for 16 h with 2 mM of racemic mixtures of 4-OH-C6 (**e**) or 4-OH-C10 (**f**). Metabolites are presented as in panels **a**, **b**

When we analyzed 3T3L1 cells incubated with 2 mM 4-OH fatty acids of different chain lengths, we observed that ACAD10 inactivation almost completely abolished production of 4-P-OH-C6-CoA. In contrast, production of 4-P-OH-acyl-CoAs was still retained for longer chain lengths (Fig. 5a, b). We reasoned that this might be due to the presence of ACAD11 in ACAD10 knockout cell lines, which might have a different substrate specificity. To test this, we produced recombinant ACAD11 and compared its function in vitro with ACAD10 in the presence of either 4-OH-C6-CoA or 4-OH-C10-CoA. Both ACAD10 and ACAD11 produced the phosphorylated products. In both cases, the presence of an intact ACAD domain led to the formation of 2-enoyl-CoAs, which was not observed when the ACAD domain was mutated in ACAD11 or left out in the case of ACAD10. To our surprise, production of 4-P-OH-acyl-CoAs and 2-enoyl-CoAs was actually comparable for ACAD11 and ACAD10 (Fig. 5c, d). This indicated that factors beyond the specificity of these enzymes contribute to the substrate utilization in cells. Usage of substrates in cells not only depends on the specificity of enzymes but also on the access of these enzymes to the substrates. ACAD10 is mitochondrial [22, 23] and ACAD11 is peroxisomal [35–38]. Thus, differences in the uptake of the substrates by these compartments and their conversion into coenzyme A adducts [39, 40] might explain why ACAD10 knockout 3T3L1 cells fail to metabolize 4-OH-C6. To explore whether this observation was unique to 3T3L1 cells, we also performed comparable experiments in HepG2 cells. In this case, inactivation of ACAD11 led to an almost complete absence for 4-P-OH-C10 whereas 4-P-OH-C6 was unaffected. In reverse, inactivation of ACAD10 led to a significant reduction of 4-P-OH-C6, whereas 4-P-OH-C10 was unaffected (Fig. 5e, f).

We conclude from these experiments that ACAD10 and ACAD11 join forces to metabolize 4-OH-fatty acids, and that the requirement of these enzymes for specific chain lengths might be influenced by their subcellular localization (Fig. 5g). Yet, further studies are needed to determine whether differences in substrate specificity exist among very long chain fatty acids, which are typically metabolized in peroxisomes.

## Discussion

While our paper was in preparation, a preprint has been deposited at BioRxiv reporting similar findings [41], corroborating that ACAD10 and ACAD11 introduce 4-hydroxyacyl-CoAs into β-oxidation. In the following we will discuss the findings obtained during our investigations.

### Introduction of 4-hydroxyacyl-CoAs into β-oxidation via enzymatic activities derived from a single polypeptide

Enzymes of the same metabolic pathway are sometimes physically linked [42]. In some instances, several enzymatic activities reside in the same polypeptide. For example, fatty acid synthase reunites all enzymatic activities required for the synthesis of palmitoyl-CoA [43]. In this case, substrates remain covalently attached to the enzyme while undergoing several enzymatic reactions. This ensures that reaction intermediates do not diffuse away, but undergo a series of sequential reactions. In other instances, multiprotein complexes organize metabolic pathways in metabolons [42, 44]. To certain extent, substrates might be directly transferred from one catalytic site to another one, but in most cases evidence for such substrate channeling is lacking. Therefore, multi-protein complexes and multi-domain proteins might simply serve to maintain a defined stoichiometry of the different enzymes within the same pathway.

ACAD10 and ACAD11 contain a kinase and an ACAD domain. Both form a 4-P-OH-acyl-CoA intermediate, which is produced by the kinase and consumed by the ACAD domain. It is tempting to speculate that the presence of both activities might ensure that the 4-P-OH-acyl-CoA intermediate is efficiently used, but this is not supported by the finding that the two domains are physically separated in vivo by enzymatic processing.

### Why could the cleavage of a bifunctional protein be useful?

ACAD10 is cleaved to produce an N-terminal and a C-terminal part, separating the kinase from the ACAD domain. We did not observe any evidence that these two parts form a stable complex in the experiments where we purified ACAD10 via N-terminal or C-terminal affinity tags. Thus, we do not have any evidence for substrate channeling. The production of a common precursor that undergoes specific cleavage might seem unnecessarily complicated. Yet, it represents a reliable means to coordinate the synthesis of both enzymes and maintain a consistent stoichiometry of both domains of ACAD10. A published study had reported that ACAD11 could also be cleaved during import into mitochondria [45]. Yet, this study relied on an incomplete cDNA that lacked the majority of the ACAD11 kinase domain, making these results difficult to interpret. In our experiments, we did not observe any evidence for cleavage of ACAD11.

The N-terminal part of ACAD10 contains an HAD domain which is absent in ACAD11. Thus, it was conceivable that the cleavage of ACAD10 might separate the ACAD domain not only from the kinase but also from the HAD domain. Many HAD domain proteins serve as phosphatases [21, 46]. Thus, we initially hypothesized that this domain might dephosphorylate P-OH-acyl-CoAs to limit their accumulation in cells. Using recombinant enzymes, we only observed negligible phosphatase activity. Accordingly, the HAD domain only contains one aspartate from the canonical DxD motif present in other family members [21], suggesting that this domain might serve a non-catalytic function. We consistently observed higher kinase activity when the remaining aspartate in the HAD domain was converted into alanine. This indicated that the HAD domain of ACAD10 is an allosteric domain that exerts feedback inhibition on the catalytic domain and limits the accumulation of 4-P-OH-acyl-CoAs. The cleavage of ACAD10 might allow for a regulation of the kinase function without affecting the ACAD function. The mitochondrial respiratory chain is a major source of reactive oxygen species, which lead to lipid peroxidation upstream of the formation of hydroxylated fatty acids. Future studies will need to reveal whether hitherto unknown metabolites might help coordinate the degradation of 4-OH fatty acids with mitochondrial metabolism.

### ACAD10 and ACAD11 follow an unusual catalytic mechanism

Phylogenetically, the ACAD domains of ACAD10 and ACAD11 are very distinct from other ACADs. This is best illustrated with their higher degree of sequence identity to orthologous bacterial enzymes than to all other human ACADs. In reverse, orthologs in bacteria are much closer to human ACAD10 and ACAD11, than to other bacterial enzymes. This indicates that these proteins form a distinct subgroup within the ACAD family.

When ACADs introduce a double bond into their substrates, electrons are transferred to FAD and subsequently to electron-transferring flavoprotein (ETF). In vitro assays therefore rely on the presence of an artificial electron acceptor. We observed that ACAD10 and ACAD11 activity did not require the addition of electron acceptors. The most parsimonious explanation for this finding is that the electrons are transferred back onto the substrate.

The catalytic cycle of ACADs starts with the removal of a proton bound to carbon 2 [20]. This facilitates the transfer of a hydride ion from carbon 3 onto FAD. Two arguments indicate that ACAD10 follows a similar mechanism. First, when we mutate the aspartate corresponding to the residue required for the deprotonation on carbon 2 in related proteins, activity of the ACAD domain is abolished. Second, when we incubated 4-P-OH-C6-CoA with the ACAD domain of ACAD10 in the presence of deuterated water we observed evidence for a reversible deprotonation of this compound (Fig. 3e). This indicates that 4-P-OH-2-enoyl-CoA and FADH_2_ might form in the first step, but it does not explain how the cofactor FADH_2_ would be reoxidized to FAD.

ACAD10 and ACAD11 act on phosphorylated compounds. Elimination of a phosphate group to form a double bond in reaction intermediates is a catalytic strategy employed by several enzymes (see EC 4.2.3. family) [47]. Of note, in chorismate synthesis, the reversible transfer of electrons from a flavin cofactor seems to trigger the phosphate elimination [48]. In the case of ACAD10/11, the formation of a conjugated double bond system in 2,4-dienoyl-CoA would make this elimination reaction energetically favorable (Fig. 3d). The resulting 2,4-dienoyl-CoA would then need to be reduced to form a 2-*trans*-enoyl-CoA, likely via the transfer of a hydride ion from FADH_2_ onto carbon C5 similar to what has been reported to occur in *E. coli* 2,4-dienoyl-CoA reductase [49, 50] (Fig. 3d). We do not observe the postulated transient intermediates (i.e., 2,4-dienoyl-CoA and 4-P-OH-2-enoyl-CoA) suggesting that they are short-lived, rapidly turned over or tightly bound to the enzyme.

Curiously, in reactions with recombinant ACAD10 and ACAD11 we not only observed the formation of 2-*trans*-enoyl-CoAs but also 3-hydroxyacyl-CoAs, suggesting that 2-*trans*-enoyl-CoA is further hydrated. Comparable observations have previously been made with the *Pseudomonas putida* orthologs of ACAD10 and ACAD11. In addition, other ACAD enzymes seem to have some enoyl-CoA hydratase activity [51]. Yet, it is still unclear whether this activity is the result of an intrinsic activity of these enzymes or due to the contamination with a small amount of an enoyl-CoA hydratase.

### Low flux pathway eliminating degradation products from many other pathways?

Genome wide association studies have revealed that single nucleotide polymorphisms in ACAD10 are strongly associated with hypertension, diabetes, weight gain or glaucoma [52–56]. Yet, some of these associations might be due to the immediate proximity of ACAD10 with the gene *ALDH2*, which has been implicated by genome-wide association and by mechanistic studies in similar medical conditions [57–60]. So far, no patients with ACAD10 or ACAD11 deficiency have been described.

In the initial description of an ACAD10 knockout mouse model, diet induced obesity and insulin resistance were reported [61]. Yet, these observations were not reproduced in a follow-up study [22]. It has also been reported that ACAD10 plays an evolutionary conserved role in mediating the antiproliferative effects of metformin [23]. Yet, in a mouse model, ACAD10 deficiency does not alter the metabolic response to metformin [22].

Many different sources can contribute to the formation of 4-OH-fatty acids. Beyond intake from dietary sources and the intestinal microbiota [9–12], this includes signaling mediators produced from polyunsaturated fatty acids [2, 3]. On the other side, this includes 4-OH-nonenal, a highly reactive breakdown product of oxidized lipids [7, 8]. These compounds play important roles in human biology and aging. Yet, ACAD10 and ACAD11 do not directly act on these bio-active lipids, but on reaction intermediates that are downstream in their degradation pathway. Thus, their deficiencies are not necessarily expected to directly affect levels of bio-active lipids.

This being said, there might be specific physiological or pathological conditions where upstream metabolites may accumulate. For example, genetic defects in succinic semialdehyde dehydrogenase prevent the oxidation of succinic semialdehyde in the catabolism of the neurotransmitter gamma-aminobutyrate (GABA). This leads to a ubiquitous increase in 4-OH-butyrate [62] and to an increase in 4-P-OH-butyryl-CoA in the brain and the liver [7, 8]. Affected patients show neurodevelopmental disorders. It is tempting to speculate that part of these symptoms might be caused by 4-P-OH-butyryl-CoA or an overload of ACAD10.

Yet other conditions might exist. Levels of lipid peroxidation have been reported to be higher in many other pathological conditions like diabetes, metabolic syndrome, autism and Down’s syndrome. Furthermore, oxylipins increase during inflammatory responses and are eliminated via mitochondrial and peroxisomal β-oxidation [63].

Future studies will need to reveal to what extent ACAD10 and ACAD11 might act as disease modifiers under these conditions. A partial redundancy in their function will likely make these explorations difficult.

## Materials and methods

### Plasmids

Guide RNAs targeting human or mouse ACAD10 and ACAD11 were inserted into the BbsI site of the vector pX458 [64]. Primer sequences are listed in Table S1.

To re-express ACAD10 or ACAD11 in knockout cell lines, we used lentiviral vectors based on the plasmid pLVX-PURO (Clontech/Takara) which expressed the puromycin resistance gene under the control of the PGK promoter. Gene expression is either driven by the CMV promotor (pUB83) or the SV40 promoter (pUB82).

To overexpress proteins in HEK293 cells we used the lentiviral vector pOH233-1 (containing a C-terminal SFB tag consisting of an S-tag, two FLAG tags and a streptavidin-binding peptide) [65] or the equivalent containing a N-terminal SFB tag (pOH147). Site-direct mutagenesis was performed by inserting two overlapping PCR products via Gibson assembly (Hifi Assembly Master Mix, New England Biolabs) [66]. Constructs for the expression of bacterial proteins were generated using a synthetic geneblock (IDT) as a template. Primers and Plasmids are presented in Tables S1 and S2, and detailed maps are available upon request.

### Cell culture

HepG2 human hepatoblastoma, HEK293 and HEK293T human embryonic kidney cell lines (gifts from Eric R. Fearon, University of Michigan) were cultured in DMEM (Biowest) supplemented with 10% fetal bovine serum (FBS) (Cytiva), 1 mM Ultraglutamine (Lonza), 100 µg/mL streptomycin, and 100 U/mL penicillin (100 U/mL) at 37 °C in the presence of 5% CO_2_ and at 100% humidity. Mouse 3T3L1 preadipocytes were grown in the same medium but with 10% newborn calf serum instead of FBS.

Recombinant lentiviruses were produced by transient transfection of HEK293T cells with lentiviral vectors and second generation packaging plasmids psPAX2 and pMD2.G (kind gifts of Didier Trono, Addgene #12260 and #12259) as described before [67]. Culture medium was changed 6 h after transfection, and recombinant viruses were recovered in the culture supernatant 24 h later. Target cells were transduced by incubation with virus-containing culture supernatant in the presence of 4 μg/mL polybrene (Sigma). Infected cells were selected 24 h later for 4 days with puromycin (Thermofisher) at concentrations of 1.5 μg/mL for HepG2 and HEK293 cells and 1 µg/mL for 3T3L1 cells.

To generate knockout cell lines, cells were transfected in 6-well plates with a 2 µg CRISPR/Cas9—guide-RNA expression plasmids and 4 µL lipofectamine 2000 following the manufacturer’s instructions (Life Technologies). Transfected cells were selected by flow cytometrical sorting gating for GFP fluorescence on a FACSAria III flow cytometer. The specific combination of guide RNAs and vector systems used for individual clones is listed in table S1 and S2. Individual clones were expanded and analyzed by Sanger sequencing (Eurofins).

### Quenching and metabolite extraction

Metabolomic analyses were performed essentially as previously reported [68]. Cell lines were plated in 6-well plates (HepG2) or 6 cm dishes (3T3L1). Lysates were obtained after quenching of metabolism with liquid nitrogen as described before (12). Briefly, culture plates were submerged in liquid nitrogen after one rapid wash with ice-cold water. 250 μL of a solution consisting of 90% methanol (Biosolve) and 10% chloroform (Fisher Scientific) was added and lysates were transferred into microcentrifuge tubes. After centrifugation for 15 min at 4 °C and 22 000 g, the supernatant was recovered, dried in a SpeedVac vacuum concentrating system (Life Technologies) and resuspended in 35 μL of 1:1 methanol:water before analysis.

To quench enzymatic reactions, we added 4 volumes of ice-cold methanol, followed by centrifugation to precipitate proteins. The supernatant was recovered for LC–MS analysis and brought to 50% methanol.

### Quantification of metabolites by mass-spectrometry

Analyses by LC–MS were performed as previously described [68] based on a method by Coulier and colleagues [69]. Briefly, 5 μL of sample were analyzed with an Inertsil 3 μm particle ODS-4 column (150 × 2.1 mm; GL Biosciences) at a constant flow rate of 0.2 mL/min with an Agilent 1290 HPLC system. Mobile phase A consisted of 5 mM hexylamine (Sigma-Aldrich) adjusted to pH 6.3 with acetic acid (Biosolve) and phase B of 90% methanol (Biosolve)/10% 10 mM ammonium acetate (Biosolve) adjusted to pH 8.5 with ammonia (Merck, Darmstadt, Germany). The mobile phase profile consisted of the following steps and linear gradients: 0–2 min at 0% B; 2–6 min from 0 to 20% B; 6–17 min from 20 to 31%B; 17–36 min from 31 to 60% B; 36–41 min from 60 to 100% B; 41–51 min at 100% B; 51–53 min from 100 to 0% B; 53–60 min at 0% B.

Analytes were identified and quantified with an Agilent 6550 mass spectrometer with an electrospray ionization (ESI) source in negative mode using the following settings: ESI spray voltage 3500 V, sheath gas 350 °C at 11 L/min, nebulizer pressure 35 psig and drying gas 200 °C at 14 L/min. An m/z range from 70 to 1200 was acquired with a frequency of 1 per second by summing 8122 transients. Compound identification was based on their exact mass (< 5 ppm) and retention time compared to standards (Sigma Aldrich, and synthesized in the lab) (Supplementary Table S3). The areas under the curve (AUC) of extracted-ion chromatograms of the [M-H]^−^ forms were integrated using MassHunter Quantitative Analysis Software (Agilent, Santa Clara, CA, USA), and normalized to the mean of the areas obtained for a series of 150 other metabolites (‘total ion current’). Further normalization steps are indicated in the figure legends.

To separate hexenoyl-CoA standards, 5 µL sample were separated with a Acquity BEH Shield RP18 column (C18, 2.1 × 100 mm, 1.7 µm; Waters) column at a constant flow of 0.2 mL/min. Mobile phase A consisted of 5 mM ammonium acetate pH 8.3 and phase B of 90% acetonitrile and 10% 5 mM ammonium acetate pH 8.3. The mobile phase profile contained the following steps and linear gradients: 0–3 min at 3% B; 3–40 min from 3 to 40% B; 40–50 min from 40 to 100% B; 50–51 min from 100 to 3% B; 51–61 min 3% B.

### Affinity purification of ACAD10 with epitope tags from HepG2 cells

HepG2 cells were transduced with recombinant lentiviruses driving expression of human ACAD10 carrying an N-terminal HA-tag and a C-terminal streptavidin-binding peptide (SBP). Cell lines were selected with puromycin. Subsequently, cells were washed with phosphate-buffered saline (PBS), removed from plates in PBS using a cell scraper, and collected by centrifugation at 400 g and 4 °C. The pellets were resuspended in 300 µL lysis buffer (150 mM NaCl, 1 mM EDTA, 20 mM Tris–HCl pH 8.0, 0.2% NP40/Igepal CA-630) per subconfluent 100 mm plates. After sonication, lysates were centrifuged at 27,000 g for 15 min at 4 °C.

To purify ACAD10 via the N-terminal HA-tag, we used Thermo Scientific ™ Pierce™ anti-HA Magnetic beads. Briefly, a protein quantity of 16 mg was applied to 50 uL beads. After rotation for 1 h at 4 °C, the solution was centrifuged at 4 °C and 400 g and the supernatant was removed. Beads were washed four times in 1 ml lysis buffer and the pellet was stored at -80 °C. Purification of ACAD10 via a C-terminal SBP was performed as described below.

### Proteomic analysis

16 mg of proteins were used as starting material to pulldown tagged proteins. 50 µL of Streptavidin Sepharose High-Performance beads or Thermo Scientific™ Pierce™ Anti-HA Magnetic Beads were used, accordingly. After purification (see above), beads were resuspended in 50 µL of a 5% SDS solution containing 50 mM Tris pH 8.5 and 5 mM DTT. After 15 min at 55 °C, chloroacetamide was added to a final concentration (f.c.) of 20 mM followed by another 10 min incubation at room temperature. Samples where then acidified by addition of phosphoric acid to a f.c. of 2.5%. 25 µL of each sample were transferred onto a micro S-Trap column (Protifi LLC, USA). Digestion was performed at 37 °C overnight with a 1:100 ratio of trypsin and LysC. Digested peptides were eluted in three steps using 40 µL each of 50 mM Tris pH 8.5, 0.2% formic acid and 50% acetonitrile. The eluted peptides were dried down in a vacuum concentrator (SpeedVac, Thermo Scientific) and resuspended in 2% Acetonitrile and 0.2% formic acid. Peptide concentration was determined by Pierce™ Quantitative Peptide Assay (Thermo Scientific). Peptide separation was performed using a reversed-phase analytical column (EasySpray, 0.075 × 250 mm, Thermo Scientific) with a linear gradient of 4–27.5% solvent B (0.1% FA in 80% ACN) for 37 min, 27.5–50% solvent B for 20 min, 50–95% solvent B for 10 min at a constant flow rate of 300 nL/min on a Vanquish Neo HPLC system. The peptides were analyzed by an Orbitrap Fusion Lumos tribrid mass spectrometer with an ESI source (ThermoFisher Scientific) coupled online to the nano-LC. Peptides were detected in the Orbitrap at a resolution of 120,000. Peptides were selected for MS/MS using the HCD setting at 30; ion fragments were detected in the Orbitrap at a resolution of 30,000. A data-dependent procedure alternating between one MS scan followed by MS/MS scans was applied for 3 s for ions above a threshold ion count of 50,000 in the MS survey scan with 20.0 s dynamic exclusion. The electrospray voltage applied was 2.1 kV. MS1 spectra from m/z 300 to 1800 were obtained with an AGC target of 400,000 and a maximum injection time set to custom. MS2 spectra were acquired with an AGC target of 10,000 and a maximum injection time set to custom.

The resulting MS/MS data were processed using the Sequest HT search engine within Proteome Discoverer 2.5 SP1 against a database containing only ACAD10 corresponding sequences (wild-type full length containing HA and Streptavidin tags and a shorter version containing the C-terminus). Trypsin or GluC was specified as cleaving enzyme in a setting were a specific cleavage only on one side was required, allowing up to 2 missed cleavages, 4 modifications per peptide and up to 7 charges. Mass error was set to 20 ppm for precursor ions and 0.05 Da for fragment ions. Oxidation on Met (+ 15.995 Da) and Methionine loss (− 131.040 Da) on the N-terminus of the protein and peptides were considered as variable modifications, whereas carbamidomethylation of cysteine was considered as a fixed modification. The False discovery rate (FDR) was assessed using the target decoy PSM validator node, and thresholds for the identification of proteins, peptides and modification sites were specified at 1%. Label-free quantification of peptides is based on the precursor ion intensity. Signals were normalized to the sum of all signals within each individual sample. Protein abundances were calculated as the sum of the abundances of unmodified peptides. Raw and processed mass spectrometry proteomics data have been deposited to the ProteomeXchange Consortium via the PRIDE partner repository with the dataset identifier PXD050316.

### Western blot analysis

Western Blot analyses were performed as described before [70]. Briefly, cell lysates were prepared in in RIPA buffer [150 mM NaCl, 20 mM Tris/HCl (pH 7.5), 1% Nonidet P40, 0.5% sodium deoxycholate and 0.1% SDS] containing a Complete™ proteinase inhibitor cocktail (Merci), followed by sonication. Tisues were powdered in liquid nitrogen prior to homogenization and sonication. Protein concentration was determined using the BCA assay and equal amounts were resolved on 10% polyacrylamide gels and transferred on to PVDF membranes (Millipore) using tank-transfer system (Bio-Rad). Membranes were blocked for 1 h with 5% non-fat dried skimmed milk powder in TBST (Tris-buffered saline containing 0.1% Tween) at room temperature (22 °C). Incubations with primary antibodies were performed overnight at 4 °C in Tris-buffered saline containing 2% BSA. Antibody concentrations were 1:1000 for ACAD10 (17161-1-AP, Proteintech), 1:5000 anti-FLAG (M2, Sigma), 1:2000 anti-HA (C29F4, Cell signaling) and 1:5000 anti-β-actin (Sigma). Subsequently, membranes were washed and incubated in horseradish peroxidase (HRP)-coupled secondary antibodies in TBST containing 5% non-fat dried skimmed milk powder. Signals were revealed using chemiluminescent HRP substrates (Milipore) and detected using a chemiluminescent detection system (Cytiva). Quantifications were performed using Imagequant TL (Cytiva). Presented values represent data from at least three independent experiments.

### Prediction of cleavage site for mitochondrial processing peptidase

To predict internal mitochondrial cleavage sites with algorithms designed to detect N-terminal mitochondrial targeting sequences [28, 29], we submitted a series of progressive N-terminal one amino acid deletions to the prediction servers and noted the predicted existence and identity of the cleavage site.

### Purification of recombinant ACAD10 and ACAD11

HEK293 cells were infected with recombinant lentivectors driving expression of full length or fragments from ACAD10 or ACAD11. Stable cell lines were selected with Puromycin. After two washes with phosphate-buffered saline, cells were removed from plates in PBS using a cell scraper and collected by centrifugation. Pellets were resuspended in 4 volumes of lysis buffer [150 mM NaCl, 1 mM EDTA, 20 mM Tris–HCl, pH 8.0, 1% NP40/Igepal CA-630, 1 µg/mL leupeptin, 1 µg/mL aprotinin] with 5 µM FAD. After sonication, lysates were clarified by centrifugation at 27,000 g for 15 min at 4 °C. We added 100 μL of streptavidin Sepharose beads (GE healthcare) per twenty 100 mm plates. After rotation for 1 h at 4 °C, beads were washed four times in lysis buffer, followed by one wash with 1 mL buffer containing 150 mM NaCl and 20 mM Tris, pH 8). Proteins were frozen down at -80 °C bound to beads in aliquots corresponding to two 100 mm plates. Before enzymatic reactions, pellets were resuspended in 25 mM Tris–Cl pH 7.4. Protein concentrations were determined by comparing band intensities with those obtained for bovine serum albumin in polyacrylamide gels stained with Coomassie blue (Pageblue, Fisher).

### Purification of bacterial enzymes

The expression construct for FadD was obtained from the ASKA collection [71], whereas the other ones are described above (see also table S2). Expression plasmids were transformed into the *E. coli* BL21 Rosetta (DE3) strain using electroporation.

For the expression of *Pseudomonas putida* LvaE and the *Burkholderia pseudomallei* ortholog of LvaA and LvaC, overnight cultures were diluted 1:50 in 100 to 500 mL Lysogeny broth (LB) containing the required antibiotics (30 µg/mL kanamycin or 100 µg/mL ampicillin). Cultures were incubated at 37 °C while shaking at 200 rpm until the optical density at 600 nm reached 0.5. Expression was induced with 1 mM isopropyl-β-D-thiogalactopyranoside (IPTG) followed by an incubation at 16 °C for 18 h with agitation at 200 rpm. Bacteria were collected by a 20 min centrifugation at 5000 × *g* and at 4 °C. Pellets were stored at − 80 °C until purification.

For the expression of His-tagged *E. coli* FadD, an overnight culture at 37 °C was diluted 1:200 in fresh LB containing 30 µg/mL chloramphenicol. The culture was incubated at 20 °C until the optical density at 600 nm reached 0.5. Expression was then induced with 1 mM isopropyl-β-D-thiogalactopyranoside (IPTG) followed by an incubation at 20 °C for 4 h with agitation at 200 rpm. Bacteria were collected by a 20 min centrifugation at 5,000 × g and 4 °C. Pellets were stored at − 80 °C until purification.

Purification His_6_-tagged *Pseudomonas putida* LvaE (Q88EH6) and the *Burkholderia pseudomallei* ortholog (Q63VL0) of LvaA (Q88G01) was adapted from Rand et al. [18]. Briefly, frozen pellets were thawed on ice and resuspended in lysis buffer containing 50 mM Na_2_HPO_4_, 300 mM NaCl, 10 mM imidazole, 2 mM DTT, pH 8.0 and 2 µL of benzonase. Cell suspensions were lysed by three cycles of sonication and 2 passages through a French press, followed by centrifugation at 25,000 × g and 4 °C for 30 min. The supernatant was filtered through a 0.45 µm filter (Sartorius). An AKTA liquid chromatography system (Cytiva) was used at a constant flow rate of 1 mL/min. A 1 mL HisTrap HP column was equilibrated with 5 column volumes (CV) of wash buffer [50 mM Na_2_HPO_4_, 300 mM NaCl, 40 mM imidazole, 2 mM DTT, pH 8.0]. After sample application, the column was washed with 15 CV wash buffer, followed by protein elution using a linear gradient reaching 100% elution buffer [50 mM Na2HPO4, 300 mM NaCl, 250 mM imidazole, 2 mM DTT, pH 7.8] over the course of 20 min, collecting 1 mL fractions. A G25 Sepharose column (PD-10 desalting, Cytiva) was used to remove imidazole after equilibration with a buffer containing 100 mM NaCl, 2 mM MgCl_2_ and 2 mM DTT. Absorbance at 280 nm was measured, and protein concentrations were computed using extinction coefficients calculated with the ProtParam tool on the ExPASy server. Protein was stored at − 80 °C until use.

For purification of the MBP-tagged LvaC ortholog from *Burkholderia pseudomallei* (Q63VL1), pellets were resuspended in 50 mM Tris–HCl pH 7.5, 50 mM NaCl, 5 mM EDTA, 2 mM β-mercaptoethanol, 2 mM MgCl2, 1 mg/mL lysozyme, 1 µg/mL aprotinin, 1 µg/mL leupeptin, and 2 mM p-toluene-sulfonyl fluoride. Cell lysis and recovery of soluble proteins was achieved as described above. The supernatant was incubated with 1 mL of prewashed amylose beads (E8021S, New England Biolabs) for 1 h on a rotating device at 4 °C. The bead suspension was spun down at 400 g for 5 min and supernatant was removed. The beads were resuspended in 5 mL lysis buffer and transferred into a 10 mL disposable column polyethylene filter (Thermo Scientific). The column was washed with 10 mL lysis buffer adjusted to 200 mM NaCl (wash buffer). Proteins were eluted by 9 successive additions of 500 µL elution buffer [wash buffer with 10 mM maltose]. To enhance elution, the column outflow was blocked for 15 min after addition of the first portion.

For purification of His-tagged *E. coli* FadD, frozen pellets were thawed on ice and resuspended in lysis buffer containing 50 mM Na_2_HPO_4_ pH 7.5, and 300 mM NaCl. Cell suspensions were lysed by three cycles of sonication, followed by centrifugation at 25,000 × g and 4 °C for 30 min. An AKTA liquid chromatography system was used at a constant flow rate of 1 mL/min. 1 mL HisTrap HP column was equilibrated with 10 column volumes (CV) of wash buffer [50 mM Na_2_HPO_4_, 300 mM NaCl, 10%glycerol]. After sample application, the column was washed with 30 CV wash buffer, followed by protein elution with 20 CV using a linear gradient from 2 to 100% elution buffer [50 mM Na_2_HPO_4_, 300 mM NaCl, 500 mM imidazole, 10% glycerol] over the course of 20 min, collecting 1 mL fractions. Two 1 ml fractions were dialyzed against lysis buffer to remove imidazole, using a Spectra/Por® Dialysis membrane with a 3 kDa molecular weight cut-off.

### Synthesis of substrates

The 4-OH, 5-OH and 6-OH fatty acids used in this study were obtained after opening γ, δ, ε-lactones respectively (Sigma 303836, 389579, 704067, V403, W279609, W278106, W253901, D804). Saponification of lactones was performed as follows: 50 mM NaOH was added to a same volume of 50 mM lactone. The mixture was heated at 90 °C for 10 min. After cooling to room temperature, the solution was neutralized using HCl.

Production of R-4-OH-C6-CoA and S-4-OH-C6-CoA occurred in 1 ml reactions, with 10 mM R-4-OH-C6 (Sigma 75378) or S-4-OH-C6 (Sigma 77011), 8.5 ng/μL LvaE, 2.5 mM CoA, 2 U/mL inorganic pyrophophatase (Roche), and a buffer consisting of 25 mM TrisHCl pH 7.4, 10 mM ATP, 10 mM MgCl2, 200 µM TCEP, 0.1 μg/μL BSA. After 1 h incubation at 37 °C, reactions were stopped in liquid nitrogen, and stored at − 80 °C until purification.

Different isomers of hexenoyl-CoA were synthesized as described above for 4-OH-C6-CoA, but using the corresponding hexenoic acid as substrate (*trans*-2-hexenoic acid—Sigma W316903, *cis*-3-hexenoic acid Sigma-PH018432, *trans*-3-hexenoic acid Sigma -W317004, and a mixture of *cis*-4-hexenoic acid and *trans*-4-hexenoic acid -Santa Cruz CAS 35194-36-6) with recombinant LvaE.

R-4-OH-C6-CoA and S-4-OH-C6-CoA were purified by preparative HPLC on a Waters systems and characterized by MS. The column used was an XBridge Prep C18 19 × 150 mm reverse-phase chromatography column (Waters). A binary solvent system was used in a 30-min linear gradient of acetonitrile in water (3–18%) containing 0.1% TFA at a flow rate of 19 ml/min. The lyophilized products were diluted to a final concentration of 10 mM in Milli-Q water and stored at − 80 °C.

### Enzymatic reactions

To assess ACAD10 and ACAD11 function, 4-OH-C6-CoA and 4-OH-C10-CoA were synthesized in situ using recombinant LvaE (*Pseudomonas putida* acyl-CoA synthetase) or FadD (*E. coli* acyl-CoA synthetase), respectively. Briefly, LvaE or FadD (8.5 ng/μL) was incubated in a 30 µL reaction with substrate 2.75 mM 4-OH-C6 (Sigma 303836) or 4-OH-C10 (Sigma W236004), 0.55 mM CoA (Sigma C3144) and 2.6 U/mL inorganic pyrophosphatase (Roche 10108987001) in a buffer containing 25 mM Tris–HCl pH 7.4, 2.75 mM ATP, 2.75 mM MgCl_2_, 200 µM TCEP, 0.05 μg/µL bovine serum albumin (BSA) and 300 µM FAD. Purified wild-type and mutant ACAD10 were used at a concentration of 50 nM (Fig. 3A, B) or 25 nM (Fig. 5C, D). The bacterial orthologs from *Burkholderia pseudomallei* were used at 50 nM (Fig. 3B). After 1 h incubation at 37 °C, 10 µL aliquots of each reaction were collected and quenched on ice in tubes containing 40 µL methanol. After 2 rounds of vortexing, the tubes were centrifuged at 15,000 g for 15 min at 4 °C. The supernatant was directly analyzed by LC–MS.

Activity on 4-OH-C6-CoA (Fig. 4A–C) was assessed in 30 µL reactions with 50 µM purified R-4-OH-C6-CoA, 25 nM (1.5 ng/µL) enzyme, 25 mM Tris–HCl pH 7.4, 2.75 mM ATP, 2.75 mM MgCl_2_, 200 µM TCEP, 0.05 μg/μL BSA. After 1 h incubation at 37 °C, 10 µL aliquots of each reaction were collected and quenched on ice in tubes containing 40 uL methanol. The tubes were centrifuged at 15,000 *g* for 15 min at 4 °C. The supernatant was directly analyzed by LC–MS.

To compare the production of 4-P-OH-C6-CoA by N-terminal WT and HAD mutant ACAD10 in a time course experiment (Fig. 4B), we incubated 25 µM purified R-4-OH-C6-CoA with 3.5 nM enzyme in 30 µL reactions. After 20 min incubation at 37 °C, 10 µL aliquots of each reaction were collected at the indicated timepoints, and quenched on ice in tubes containing 40 µL methanol. Tubes were centrifuged at 15,000 *g* for 15 min at 4 °C, and the supernatant was directly analyzed by LC–MS.

To determine the activity of the ACAD10 kinase domain at different substrate concentrations, we assessed the production of 4-P-OH-C6-CoA in 30 µL reactions containing increasing concentrations of purified R-4-OH-C6-CoA (0-10-25-50-100-200 µM) and 25 nM enzyme for 60 min at 37 °C. The experimental conditions and the quenching method were otherwise as described above.

To investigate deuterium incorporation from D_2_O into the intermediates of the ACAD10 reaction, we incubated 50 µM R-4-OH-C6-CoA with 25 nM enzyme, in the presence of 50% D_2_O. Otherwise, reaction conditions, quenching, and analysis were as described above. The abundance of the M + 1 version of 4-P-OH-hexanoyl-CoA resulting from deuterium incorporation was determined after correction for the natural isotopic distribution of carbon using the Vistaflux package within the Profinder software (Agilent).

### Statistical analysis

Statistical analyses were performed in GraphPad Prism 10. Unless stated otherwise, data present the means ± SEM of three experiments containing each 2 to 3 biological replicates. Data were analysed by one-way or two-way ANOVA followed by post-hoc testing using the Dunnet or Holm-Sidak correction [72, 73].

### Supplementary Information

Below is the link to the electronic supplementary material.

Supplementary file1 (PDF 111 KB)

Supplementary file2 (PDF 88 KB)

Supplementary file3 (PDF 90 KB)

Supplementary file4 (PDF 2861 KB)

## Data Availability

Raw and processed mass spectrometry proteomics data have been deposited to the ProteomeXchange Consortium via the PRIDE partner repository with the dataset identifier PXD050316.
