# Supplementary material for: ACAD10 and ACAD11 allow entry of 4-hydroxy fatty acids into β-oxidation
Source: Cell Mol Life Sci. 2024 Aug 22;81(1):367. doi: 10.1007/s00018-024-05397-8 (PMC11342911; doi:10.1007/s00018-024-05397-8)
Supplement: Supplementary file 1 — Supplementary file1 (PDF 111 KB) [file 18_2024_5397_MOESM1_ESM.pdf]

Paquay et al, Supplementary Table S1

Table S1. Primers used in this study

Pair n°	Plasmid name	Forward primer	Intermediate primer	Reverse primer
1	ACAD10 WT in pUB83 (pFC18)	ATACATGCGGCCGCACCATGTGTGTCAGGAGCTGTT		TTATAATGTACACTAAATGCGGTGCTTCAGCTCTA
2	ACAD10 HAD mutant in pUB83 (pST24)	ATTACAGGGACAGCAGAGATCCAGTTTATC	TACAGAGCGGTGATTTTC**GCC**ATGGGCGGAGTTCTC	TTATAT TGTACA CTAAAT GCGGTG CTTCAG CTCTAG CTTG
3	ACAD10 kinase mutant in pUB83 (pST26)	TCTAGCTAGAGGATCCTTGGGT	ACAGTGGTGCACGGG**GCC**TTCAGGCTCGACAACCT	AGCGGTGCTGTCCATCTGCA
4	ACAD10 ACAD mutant in pUB83 (pST28)	AAGAGATGCCCTTCACAAATC	TGCGCTTTG CC**GCC**GGCCCTGACGAGGTGCA	AGCGGTGCTGTCCATCTGCA
5	LvaE in Pet28a (pST45)	ATACATTAATACGACTCACTATAG		TTATAT CAGGAA ACAGCTATGACCT
6	Burkhol. Kinase in Pet28a (pST43)	ATACAT CATATG GCT ACCG		ATGTAT CTC GAGTTAGCGGGC
7	Burkhol. ACAD in pet28a (pAP9)	ATACAT GGATCC ATG CAC TTCGAC TATTCT GCT		TTATAT GAATTC TAGGCC GAACGT GGC GCG TGAC
8	ACAD10 WT in pOH233 (pJG397)	ATACATGCTAGCCACCATGTGTGTCAGGAGCTGT		ATACATTGTACAAATGCGGTGCTTCAGCTCTAG
9	ACAD10 ACAD mutant in pOH233 (pJG398)	ATACATGCTAGCCACCATGTGTGTCAGGAGCTGT		ATACATTGTACAAATGCGGTGCTTCAGCTCTAG
10	ACAD10 M608 WT in pOH233 (pST67)	ATACAT GCTAGC CACCAT GTGTGT CAG		TTATAT TGTACA CATCTC TTTGAA AAC
11	ACAD10 M608 HAD mut in pOH233 (pST69)	ATACAT GCTAGC CACCAT GTGTGT CAG		TTATAT TGTACA CATCTC TTTGAA AAC
12	ACAD11 WT in pOH147 (pST73)	ATACAT GCTAGCCACC ATGAAG CCAGGTGCTACT		TTATAT TGTACA GAGAC GTTATA TCTTGGCTGTCAGTCT
13	ACAD11 ACAD mutant in pOH147 (pST75)	ATACAT GCTAGCCACC ATGAAG CCAGGTGCTACT		TTATAT TGTACA GAGAC GTTATA TCTTGGCTGTCAGTCT
14	MTSCox8-2xHA Tag ACAD10-SFB Tag in Pub82 (pJD7)	ATACAT AGATCTCGAGGGATTGCGGCCGCTGGGTCCCACCGATGGACACA		TTATATGAATTCTAGGGCTCCCGCTGGCCCTGA
15	ACAD10 deletion 1 (32AA) in pJD7 (pJD20)	CACTCGAAGACCGGTGCCAC (PCR1)<br>GAAGGGTTCAGTTATAGCTCCGTTCCAGA (PCR2)		CGGAGCTATAACTGAACCCTTCTTTGACTGCGA (PCR1)<br>GGAAAAGCGCCTCCCCTACC (PCR2)
16	ACAD10 deletion 2 (53 AA) in pJD7 (pJD21)	CACTCGAAGACCGGTGCCAC (PCR1)<br>AGAAGGGTTCCCAGAGAGCCTCTCTCCACCTGTC (PCR2)		AGAGAGGCTCTCTGGGAACCCTTCTTTGACTGCGA (PCR1)<br>GGAAAAGCGCCTCCCCTACC (PCR2)
17	ACAD10 permutation 1 in pJD7 (pJD22)	CACTCGAAGACCGGTGCCAC (PCR1)<br>CAAAGAAGGGTTCAGTTA CTCATCAGTCCCTGA (PCR2)<br>CATCTCCTGCTAGTTAT AGCTCCGTTCCAGA (PCR3)		CTGATGAGTAACTGAACCCTTCTTTGACTGCGA (PCR1)<br>GAACGGAGCTATAACTAGCAGG AGATGCTTCCGGCA (PCR2)<br>GGAAAAGCGCCTCCCCTACC (PCR3)
18	ACAD10 permutation2 in pJD7 (pJD23)	CACTCGAAGACCGGTGCCAC (PCR1)<br>CAAAGAAGGGTTCAGG AGTTACTCATCAGTCCC (PCR2)<br>AAGCATCTCCTAGTT ATAGCTCCGTTCCAGA (PCR3)		ATGAGTAACTCC TGAACCCTTCTTTGACTGCGA (PCR1)<br>GAACGGAGCTATAACT AGGAGATGCTTCCGGCA (PCR2)<br>GGAAAAGCGCCTCCCCTACC (PCR3)
19	ACAD10	CACTCGAAGACCGGTGCCAC (PCR1)		GTGATGT ATGCGCGAACCCTTCTTTGACTGCGA (PCR1)

	permutation 3<br>in pJD7 (pJD24)	CAAAGAAGGGTTCG CGCATACATCACGGGGAGGT (PCR2)<br>TGGTAATATCCAGTT ATAGCTCCGTTCCAGA (PCR3)	GAACGGAGCTATAACTGGAT ATTACCAGCCCGCCCCT (PCR2)<br>GGAAAAGCGCCTCCCCTACC (PCR3)
20	ACAD10 random<br>unstructured<br>loop in pJD7 (pJD25)	CACTCGAAGACCGGTGCCAC (PCR1)<br>CGCAGTCAAAGAAGGGTTCTCGGGGTCAGGATCCGGCGC (PCR2)<br>AGCGGAGCTATGAGTTATAGCTCCGTTCCAGAAGCTTCCCCAG (PCR3)	GACCCCGAGAACCCTTCTTTGACTGCGAA (PCR1)<br>ACGGAGCTATAACTCATAGCTCCGCTCCCACTAC (PCR2)<br>GGAAAAGCGCCTCCCCTACC (PCR3)
21	ACAD10 substit.<br>with α helix<br>in pJD7 (pJD26)	CACTCGAAGACCGGTGCCAC (PCR1)<br>AGAAGGGTTCGGCATTCTCGACACCAGGCT (PCR2)<br>AACCCGTCAGTTATAGCTCCGTTCCAGA (PCR3)	GTGTCGAGAATGCCGAACCCTTCTTTGACTGCGA (PCR1)<br>TGGAACGGAGCTATAACTGACGGGTTTTTGTTGTGTG (PCR2)<br>GGAAAAGCGCCTCCCCTACC (PCR3)
22	ACAD10<br>R603A in pJD7<br>(pJD29)	CACTCGAAGACCGGTGCCAC (PCR1)<br>TCTAACCTGGCGTGGGATTT (PCR2)<br>TGTCAGAGAGCTGTATCACC (PCR3)	CTGCGAAATCCCACGCCAGG (PCR1)<br>CAGCCGGTGATACAGCTCTC (PCR2)<br>GGAAAAGCGCCTCCCCTACC (PCR3)
23	ACAD10 R616A<br>in pJD7<br>(JD31)	CACTCGAAGACCGGTGCCAC (PCR1)<br>TCTAACCTGGCGTGGGATTT (PCR2)<br>TGTCAGAGAGCTGTATCACC (PCR3)	CTGCGAAATCCCACGCCAGG (PCR1)<br>CAGCCGGTGATACAGCTCTC (PCR2)<br>GGAAAAGCGCCTCCCCTACC (PCR3)
24	ACAD10 R634A<br>in pJD7<br>(pJD32)	CACTCGAAGACCGGTGCCAC (PCR1)<br>TCTAACCTGGCGTGGGATTT (PCR2)<br>TGTCAGAGAGCTGTATCACC (PCR3)	CTGCGAAATCCCACGCCAGG (PCR1)<br>CAGCCGGTGATACAGCTCTC (PCR2)<br>GGAAAAGCGCCTCCCCTACC (PCR3)
25	ACAD10 triple<br>mutant in pJD7<br>(pJD30)	CACTCGAAGACCGGTGCCAC (PCR1)<br>TCTAACCTGGCGTGGGATTT (PCR2)<br>TGTCAGAGAGCTGTATCACC (PCR3)	CTGCGAAATCCCACGCCAGG (PCR1)<br>CAGCCGGTGATACAGCTCTC (PCR2)<br>GGAAAAGCGCCTCCCCTACC (PCR3)
26	ACAD10 4x<br>Mutant in pJD30<br>(pJD19)	CACTCGAAGACCGGTGCCAC (PCR1)<br>CCACACGTGGGCCGCGCCCCAGTCCCAGTGGTGCC (PCR2)	GACTGGGGCGCGGCCCACGTGTGGTAGGAC (PCR1)<br>GGAAAAGCGCCTCCCCTACC (PCR2)

Target n°	Name	Target sequence	Oligonucleotides	
1	mouse ACAD10<br>CRISPR guide 1 (pST20)	CCAGCCTGATGTAGTACGTGTTT	CACC GGCCAGCCTGATGTAGTACGT	AAAC ACGTACTACATCAGGCTGGCC
2	mouse ACAD10<br>CRISPR guide 2 (pST21)	TCCCCGAGGGTTTCTTCCTCGTTT	CACC GGTCCCCGAGGGTTTCTTCCTC	AAAC GAGGAAGAAACCCTCGGGGACC
3	human ACAD10<br>CRISPR guide 1 (pST54)	AAATGAGTCCACA GGCACGG	CACC GGAAATGAGTCCACAGGCACGG	AAAC CCGTGCCTGTGGACTCATTTCC
4	human ACAD10<br>CRISPR guide 2 (pST55)	CAGTTCCCAGTGATGACTG	CACC GGCAGTTCCCAGTGATGACTG	AAAC CAGTCATCACTGGGAACTGCC
5	human ACAD11<br>CRISPR guide 1 (FC13)	GGCTTTCAAACATATGTGCTC	CACC GGGCTTTCAAACATATGTGCTC	AAAC GAGCACATATGTTTGAAAGCCC
6	human ACAD11<br>CRISPR guide 2 (FC14)	GGTGCTTTAGGAAGAAGTGAACC	CACC GGTGCTTTAGGAAGAAGTGAACC	AAAC GGTTCACTTCTTCCTAAAGCACGG
