# Supplementary material for: ACAD10 and ACAD11 allow entry of 4-hydroxy fatty acids into β-oxidation
Source: Cell Mol Life Sci. 2024 Aug 22;81(1):367. doi: 10.1007/s00018-024-05397-8 (PMC11342911; doi:10.1007/s00018-024-05397-8)
Supplement: Supplementary file 2 — Supplementary file2 (PDF 88 KB) [file 18_2024_5397_MOESM2_ESM.pdf]

Paquay et al, Supplementary Material

Table S2. Plasmids used in this study

Plasmid n°	Name	Template
1	ACAD10 WT<br>in pUB83 (pFC18)	Amplification from human ACAD10 inpEF6/Myc His A
2	ACAD10 HAD<br>mutant in pUB83<br>(pST24)	Amplification from plasmid n°1
3	ACAD10 kinase<br>in pUB83 (pST26)	Amplification from plasmid n°1
4	ACAD10 ACAD<br>mutant in pUB83<br>(pST28)	Amplification from plasmid n°1
5	LvaE in Pet28a (pST45)	Amplification from genblock sequence :<br><br>TAATACGACTCACTATAGGCCATGGGCCATCATCACCATCATCATGGGAGTATGATGGTT<br>CCGACATTAGAGCACGAGTTGGCGCCTAACGAAGCGAACCACGTTCCGTTAAGCCCCTT<br>GAGCTTCCTGAAGCGTGCCGCACAGGTGTATCCCCAACGCGACGCCGTAATCTATGGAG<br>CCCGCCGTTACTCTTACCGCCAATTGCATGAGCGCTCACGCGCCCTGGCCTCCGCTCTTGA<br>ACGCGTGGGCGTACAACCTGGGGAACGCGTTGCAATTTTAGCACCGAATATTCCGGAGAT<br>GCTTGAAGCGCATTATGGTGTACCAGGAGCGGGCGCTGTTTTAGTGTGCATCAATATTCG<br>CCTGGAAGGTCGTTCAATTGCGTTCATTTTGCGCCACTGCGCGGCCAAAGTACTTATCTGC<br>GACCGCGAGTTTGGTGCTGTCGCTAACCAAGCTTTAGCTATGTTAGACGCACCTCCCCTTTT<br>AGTGGGTATCGACGATGACCAGGCTGAACGCGCAGACCTGGCTCATGATCTGGATTACGA<br>AGCGTTTTTAGCACAGGGAGACCCAGCTCGCCCTCTGTCCGCTCCTCAAAATGAGTGGCAG<br>TCAATCGCGATCAACTATACATCAGGTACGACGGGCGATCCTAAAGGGGTAGTTTTGCACC<br>ACCGTGGCGCTTACTTAAATGCGTGTGCCGGTGCATTAATCTTTCAGCTGGGGCCTCGTTCT<br>GTCTATTTATGGACGCTGCCAATGTTCCACTGTAATGGTTGGTCCCACACCTGGGCTGTTAC<br>TCTTAGCGGGGGGACTCACGTTTGTTTGCGCAAAGTACAACCTGATGCTATTAATGCCGCCA<br>TTGCTGAGCACGCAGTGACGCATTTATCCGCCGCCCCAGTGGTCATGTCAATGCTTATTCAT<br>GCAGAGCACGCCAGCGCACCTCCAGTACCGGTGAGTGTAATTACAGGTGGGGCGGCCCCT<br>CCTAGTGCAGTCATCGCCGCTATGGAAGCCCGCGGATTCAACATCACCCATGCCTACGGGA<br>TGACTGAATCTTATGGGCCATCTACACTTTGCCTTTGGCAGCCCGGCGTGGACGAGCTTCCT<br>TTAGAGGCACGCGCCCAATTCATGAGTCGCCAAGGTGTTGCTCACCCGCTGTTAGAGGAGG<br>CGACTGTGCTTGATACCGACACGGGACGCCCGGTCCCCGCAGACGGCTTGACTCTGGGCGA<br>ATTAGTGGTCCGTGGTAACACGGTGATGAAAGGATACCTGCATAATCCTGAGGCGACCCGT<br>GCCGCTTTGGCGAATGGATGGCTTCACACGGGTGATCTTGCAGTCTTGCATCTTGACGGCTA<br>TGTGGAGATTAAGGACCGTGCGAAAGACATCATTATCTCGGGAGGAGAGAATATTTCTTCG<br>TTAGAGATTGAGGAAGTGTTGTACCAGCATCCCGAAGTAGTTGAAGCGGCAGTCGTGGCG<br>CGTCCAGATAGTCGTTGGGGTGAGACACCTCACGCTTTCGTAACCTTACGCGCCGATGCTC<br>TGGCATCGGGTGATGATCTTGTACGCTGGTGCCGCGAGCGTTTGGCTCATTTCAAGGCTCC<br>ACGTCATGTTTCACTTGTTGATTTACCCAAGACCGCGACGGGGAAAATTCAAAAGTTCGTC<br>TTGCGCGAGTGGGCACGTCAGCAGGAAGCGCAAATCGCAGATGCCGAACACTAACTCGA<br>GGTCATAGCTGTTTCCTG
6	Burkhol. Kinase<br>in Pet28a (pST43)	Amplification from genblock sequence :<br><br>ATACATCATATGGCTACCGAACATGACCCCCAGAGTAGCCAAGCCGATTATGCTGCGTTT<br>GAGGGAACACGCGCCGTGGGCGACGCGCAACGCTTTGACGTAGATGCCCTGGCCGCCT<br>GGTTAGCGAAGCATGTTGGCTCATTTGCTGGCCCGTTAGCAGTGGAGCAGTTCAAGGGA<br>GGACAATCAAACCCTACGTTCAAGCTGGTAACCCCGGCACGCTCGTATGTCCTGCGTGC<br>TAAACCCGCGCCCGCTGCCAAATTGTTGCCCAGTGCCCACGCTATCGAACGTGAGTATCG<br>TGTCATGGCGGCGCTTGCTGGAACAGGTGTGCCTGTTGCGCCAATGCTTGCACACTGCGA<br>CGATGAATCCGTTATTGGACGTGCATTCTACGTGATGGCATTCGTTGACGGACGCGTACT<br>GTGGGACCCGAGTTTGCCGGGTATGACTCCCGCAGAACGCGGACGTCATTACGACGAGAT<br>GAACCGTGTGATTGCGGCCCTTCATTCGATTGATCCACAAGCCGTAGGACTGGCCGACTA<br>TGGTAAGCCCGGGAACTACTTAGCTCGCCAGATTGCTCGCTGGTCCAAACAGTACTTGGCG<br>TCGGAAACAGAACCAATCGACGCGATGCGTCGCCTGATTGACTGGTTGCCCCAGCATTTAC<br>GTCCCGAGTCGGGCCGCGATGCACGCGTATCAATCGTACACGGGGATTACCGTCTGGACA<br>ATTTAATTTTTGACGCGCACGCACCGCGCGTGCTGGCAGTGCTGGATTGGGAGCTGTCAAC<br>GCTGGGGGACCCTTTGGCCGACTTCGCATACCATTGTATGGCCTGGCACGTTGCCCCCGAG<br>CGTTTCCGCGGAATTGCGGGATTAGACTTGCGCTCACTTGGCATCCCTGATGAAGCCCAAT<br>ATGTAGCCCGCTACTGCGAACGTACTGGACTGACCATGCCAGAAAACTGGAACTTTTACCT<br>TGCGTACAATATGTTCCGCATTGCCGCGATTTTACAAGGTATTATGAAACGTGTCGCGGAC<br>GGAACAGCTAGTAGCGCGCAGGCATTGGACGCCGGGCGTCGTGCGCGTCCTATGGCGGA<br>ACTTGCATGGCGTTACGCCCAACATGCCCGCTAACTCGAGATACAT

7	Burkhol. ACAD<br>in Pet28a (pAP9)	Amplification from genblock sequence :<br><br>ATGCACTTCGACTATTCTGCTAAGGTGGAAGCCTTGCGCGCGCGCTTGGGTGCTTTTTTCGA<br>CGAACGTATCTACCCCAACGAACGCGCCTTCTACGAGGAAATTGCACGTAACCGTCGTGCC<br>GGGGACGCGTGGCGTCCAGTCGAATTGATTGAGACACTGAAGGCGAAGGCGCGCGCCGCA<br>GGGCTTTGGAACTTGTTCTTGCCGGATAGCGCGCGTGGTGCCGGACTGACGAATCTGGAATA<br>TGCTCCATTATGTGAGATCATGGGGCGTGTCCCGTGGGCCCCAGAAGTGTTCAATTGTAACGC<br>ACCCGATACCGGGAATATGGAAACATTAGAACGCTATGGCACCGATGCCCACAAGGCTGCTTG<br>GTTGGAGCCCTTGCTGGACGGTGTGATCCGTTCGGCGTTCCTTATGACGGAACCTGAAGTCGC<br>TTCGAGCGACGCGACCAACATTCGTACCCGCATTGAACGTGATGGGGAACATTATGTCATCAA<br>TGGACACAAGTGGTGGAGCTCCGGGGCCGGCGACCCTCGCTGCAAACTGTATATCGTCATGGG<br>CAAGACAGATCCCGATGCTCCTCGCCACGCTCAACAAAGCATGATGCTTGTACCCTCGGATGCTC<br>ACGGGGTTACAGTTCACCGCCCCCTTAATGTGTTTGGGTATGACGACGCGCCGCATGGACACAT<br>GGAAGTAACTCTGGAGAACGTGCGTGTGCCTGCGTCGAATCTGCTTCTGGGGGAAGGGCGCGG<br>CTTTGAAATTGCCCAGGGGCGCTTGGGGCCGGGTCGCATTCACCATTGCATGCGTTTAGTAGGC<br>TTGGCTGAACGCGCCTTGGAGCTTATGTGTCGTCGCGCGTCCGAGCGCATCGCCTTTGGGAAGC<br>CGGTGGCTGCGCAGACTGTTACCCAAGAACGCATCGCGGAGGCACGCTGTATGATTGAACAAG<br>CTCGCTTGCTTACCTTGAAGACAGCCTATATGATGGATACGGTCGGTAACAAGGGCGCACGTGG<br>TGAGATTGCTATGATCAAGGTGGTAGCGCCGAACATGGCCTGCCAGGTTATTGACTGGGCGATT<br>CAGGCCCACGGGGGTGGGGGAGTTTCGGATGACTTCCCTTTGGCCTATGCTTACGCGTCCGCAC<br>GCACCTTGCGTTTTGCAGATGGCCCAGATGAAGTACATCGCAACGCCATTGCAAAATTAGAACTT<br>GCGCGTCACGCGCCACGTTCGGCC
8	ACAD10 WT<br>in pOH233 (pJG397)	Amplification from plasmid n°1
9	ACAD10 ACAD<br>mutant in pOH233<br>(pJG398)	Amplification from plasmid n°4
10	ACAD10 M608<br>WT in pOH233<br>(pST67)	Amplification from plasmid n°8
11	ACAD10 M608<br>HAD mut in pOH233<br>(pST69)	Amplification from full length HAD mutant ACAD10 in pOH233 (pJG399 generated from plasmid n°2)
12	ACAD11 WT in pOH147<br>(pST73)	Amplification from ACAD11WT in pJG268 (pUB83 but expressing hygromycin resistance gene)
13	ACAD11 ACAD<br>mutant in pOH147<br>(pST75)	Amplification from ACAD11 ACAD mutant in pJG268
14	MTSCox8-2xHA Tag<br>ACAD10-SFB Tag<br>in Pub82 (pJD7)	Amplification from plasmid n°8
15	ACAD10<br>deletion 1<br>(32AA) in pJD7 (pJD20)	Amplification from plasmid n°14
16	ACAD10<br>deletion 2<br>(53 AA) in pJD7 (pJD21)	Amplification from plasmid n°14
17	ACAD10<br>permutation 1<br>in pJD7 (pJD22)	Amplification from plasmid n°14 and genblock sequence :<br><br>TCGGGGTCAGGATCCGGCGCAATGAGTGGATCTGGGTCAGGCGCGATGTCTGGCTCGGGTT<br>CAGGAGCGATGTCCGGTAGTGGGAGCGGAGCTATGAGCGGGTCCGGTAGTGGCGCCATGA<br>GTGGCAGCGGATCTGGTGCTATGTCAGGAAGTGGTTCTGGGGCAATGGGCATTCTCGACAC<br>CAGGCTGAGTGAGTTGGAGAAAAACGGTGGGGCTGCACTCGCTGTACTCGATGCTCAACAG<br>GCCCGACTTTTGGGGCAGCAAACTAGAAACGATCGGGCAATCTCTGAGGCGCGAAATAAAT<br>TGAGCTCCGTCACAGAGAGCCTTAATACAGCTCGAAACGCTCTGACAAGGGCCGAGCAACAA<br>CTCACACAACAAAAACCCGTCGGTATCTTGGATACGCGGCTCAGCGAATTAGAAAAGAATGG<br>AGGTGCGGCCCTGGCCGTGCTTGACGCCCAGCAAGCGCGCCTGCTGGGACAACAGACACGCA<br>ATGACAGGGCTATTTCCGAAGCCCGGGCGCATACATCACGGGGAGGTTTGGTTATTTCTAGTTA<br>CTCATCAGTCCCTGAGGCCAGTCCAGCACACACCTCTAGGGGCGGGCTGGTAATATCCTCTT<br>ATAGCAGTGTGCCGGAAGCATCTCCTGCT
18	ACAD10<br>permutation 2<br>in pJD7 (pJD23)	
19	ACAD10<br>permutation 3<br>in pJD7 (pJD24)	
20	ACAD10 random<br>unstructured<br>loop in pJD7 (pJD25)	
21	ACAD10 substit.<br>with α helix<br>in pJD7 (pJD26)	
