# Supplementary material for: ACAD10 and ACAD11 allow entry of 4-hydroxy fatty acids into β-oxidation
Source: Cell Mol Life Sci. 2024 Aug 22;81(1):367. doi: 10.1007/s00018-024-05397-8 (PMC11342911; doi:10.1007/s00018-024-05397-8)
Supplement: Supplementary file 3 — Supplementary file3 (PDF 90 KB) [file 18_2024_5397_MOESM3_ESM.pdf]

Table S3. m/z and retention time for selected metabolites analyzed by LC-MS

Metabolite	m/z [M-H]-	retention time (min) Hexylamine method	retention time (min) C18 method
4-OH-C6	131.0714	13	
4-OH-C6-CoA	880.1760	36.5	
3-OH-C6 CoA	880.1760	37.5	
4-P-C6	211.0376	16	
4-P-C6-CoA	960.1423	36	
2-Hexenoyl-CoA	862.1654	40	16
3-Cis-Hexenoyl-CoA	862.1654		14.5
3-Trans-Hexenoyl-CoA	862.1654		15
4-Cis/Trans-Hexenoyl-CoA	862.1654		14.8
