# Supplementary material for: ACAD10 and ACAD11 allow entry of 4-hydroxy fatty acids into β-oxidation
Source: Cell Mol Life Sci. 2024 Aug 22;81(1):367. doi: 10.1007/s00018-024-05397-8 (PMC11342911; doi:10.1007/s00018-024-05397-8)
Supplement: Supplementary file 4 — Supplementary file4 (PDF 2861 KB) [file 18_2024_5397_MOESM4_ESM.pdf]

Supplementary figures

ACAD10 and ACAD11 allow entry of 4-hydroxy fatty acids into β-oxidation

Stéphanie Paquay[1-3], Julia Duraffourd[1,2], Marina Bury[1,2], Isaac P. Heremans[1,2], Francesco Caligiore[1,2], Isabelle Gerin[1,2], Vincent Stroobant[4], Jean Jacobs[1,2], Aymeric Pinon[1,2], Julie Graff[1], Didier Vertommen[5], Emile Van Schaftingen[1,2], Joseph P. Dewulf[1,2,6], Guido T. Bommer[1,2]*
[1]Metabolic Research Group, de Duve Institute & WELRI, Université Catholique de Louvain, 1200 Brussels, Belgium

This file contains Supplementary Figures S1 to S7.

a

HAD domain of ACAD10 orthologs

Human (Q6JQN1)	STYRAVIFDMGGVLIPSPGR
Mouse (Q8K370)	RSYRAVIFDTGGVLVPSPGT
Xenopus tr. ACAD10 (F7ALM6)	KGYKAVIFDMGGVLLPSPYR
C.elegans ACAD10 (O01590)	MTIKAVIFDMGGVILPAPMH
	[hhhDxDx]*

b

Kinase domain of ACAD10/ACAD11 orthologs

Human ACAD10 (Q6JQN1)	QQRTTVVHGDFRLDNLVFHP
Human ACAD11 (Q709F0)	DNEENLIHGDFRLDNIVFHP
Mouse ACAD10 (Q8K370)	QQRTTLVHGDFRLDNLIFHP
Xenopus tr. ACAD10 (F7ALM6)	EQKTTVVHGDFRLDNLIFHP
C.elegans ACAD10 (O01590)	SGKSTIVHGDFRVDNLIIEE
Burkholderia p. (Q63VL0)	DARVSIVHGDYRLDNLIFDA
Pseudomonas p. (Q88G01)	HPRPGIVHNDYRFDNVILDA

c

ACAD domain of ACAD10/ACAD11 orthologs

Human ACAD10 (Q6JQN1)	TWARALRFADGPDEVHRATV
Human ACAD11 (Q709F0)	AITRVLRLADGPDEVHLSAI
Mouse ACAD10 (Q8K370)	GWARALRFADGPDEVHQLTV
Xenopus tr. ACAD10 (F7ALM6)	AWARALRLADGPDEVHQAAV
C.elegans ACAD10 (O01590)	VWARSLRIADGPDAVHLETI
Burkholderia p. LvaC (Q63VL1)	ASARTLRFADGPDEVHRNAI
*Pseudomonas p.*LvaC (Q88K54)	AMQRTLRLADGPDEVHRAAI

d **ACAD domain**

	ACAD10	ACAD11	*Pseudomonas LvaC*	*Burkholderia LvaC*	human MCAD	human LCAD	human VLCAD	human SCAD
human ACAD10	100							
human ACAD11	54.52	100						
Pseudomonas LvaC	62.07	56.27	100					
Burkholderia LvaC	60.69	56.46	66.58	100				
human MCAD	25.83	24.40	24.08	24.67	100			
human LCAD	23.74	22.91	23.50	23.54	31.75	100		
human VLCAD	24.52	22.83	25.31	25.95	30.86	29.65	100	
human SCAD	28.89	27.96	28.61	27.03	37.9	32.74	34.72	100

e **kinase domain**

	ACAD10	ACAD11	*Pseudomonas LvaC*	*Burkholderia LvaC*
human ACAD10	100			
human ACAD11	43.19	100		
Pseudomonas LvaC	28.61	28.78	100	
Burkholderia LvaC	51.29	37.75	29.68	100

Fig. S1 Alignment of ACAD10 and ACAD11 domains with their orthologs
a-c, Alignment of sequences from the indicated proteins using Clustal Omega [2].
d, Identity matrix of the ACAD domains from the indicated proteins when aligned via Clustal Omega. This includes human ACAD10 (Q6JQN1) amino acids 637-1059, human ACAD11 (Q709F0) amino acids 374-771, *Burkholderia pseudomallei* ACAD (Q63VL1), *Pseudomonas putida* ACAD (Q88K54), human MCAD (NP_000007.1) amino acids 23-421, human LCAD (NP_001599.1) amino acids 28-430, human VLCAD (NP_000009.1) amino acids 41-482, and human SCAD (NP_000008.1) amino acids 25-473.
e, Identity matrix of the kinase domains from the indicated proteins when aligned via Clustal Omega. This includes human ACAD10 (Q6JQN1) amino acids 249-600, human ACAD11 (Q709F0) amino acids 1-358, *Burkholderia pseudomallei* ACAD (Q63VL0), *Pseudomonas putida* ACAD (Q88G01).

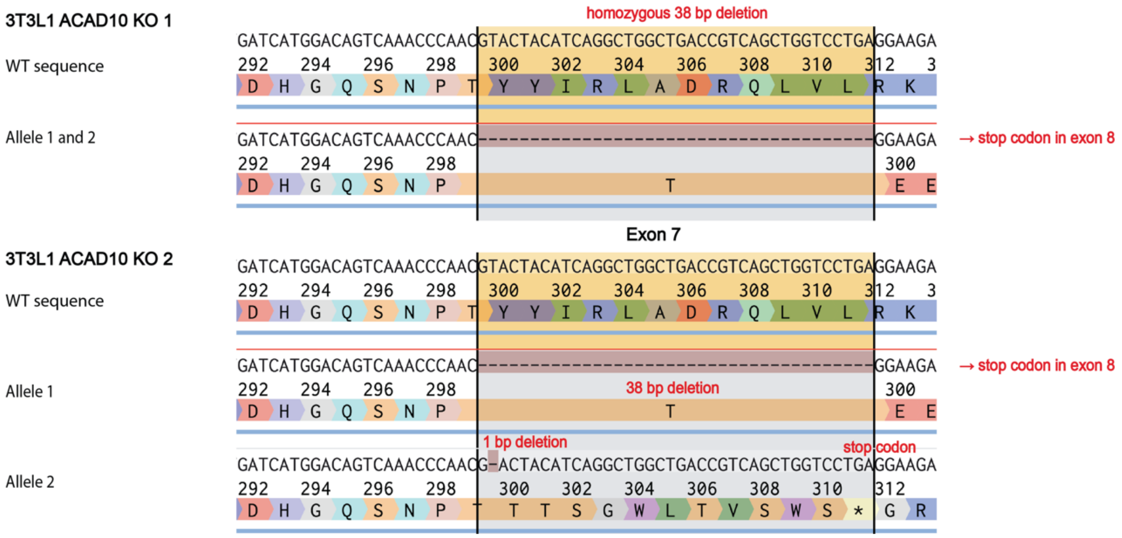

Fig. S2 Genotype of ACAD10 mutant 3T3L1 cell lines

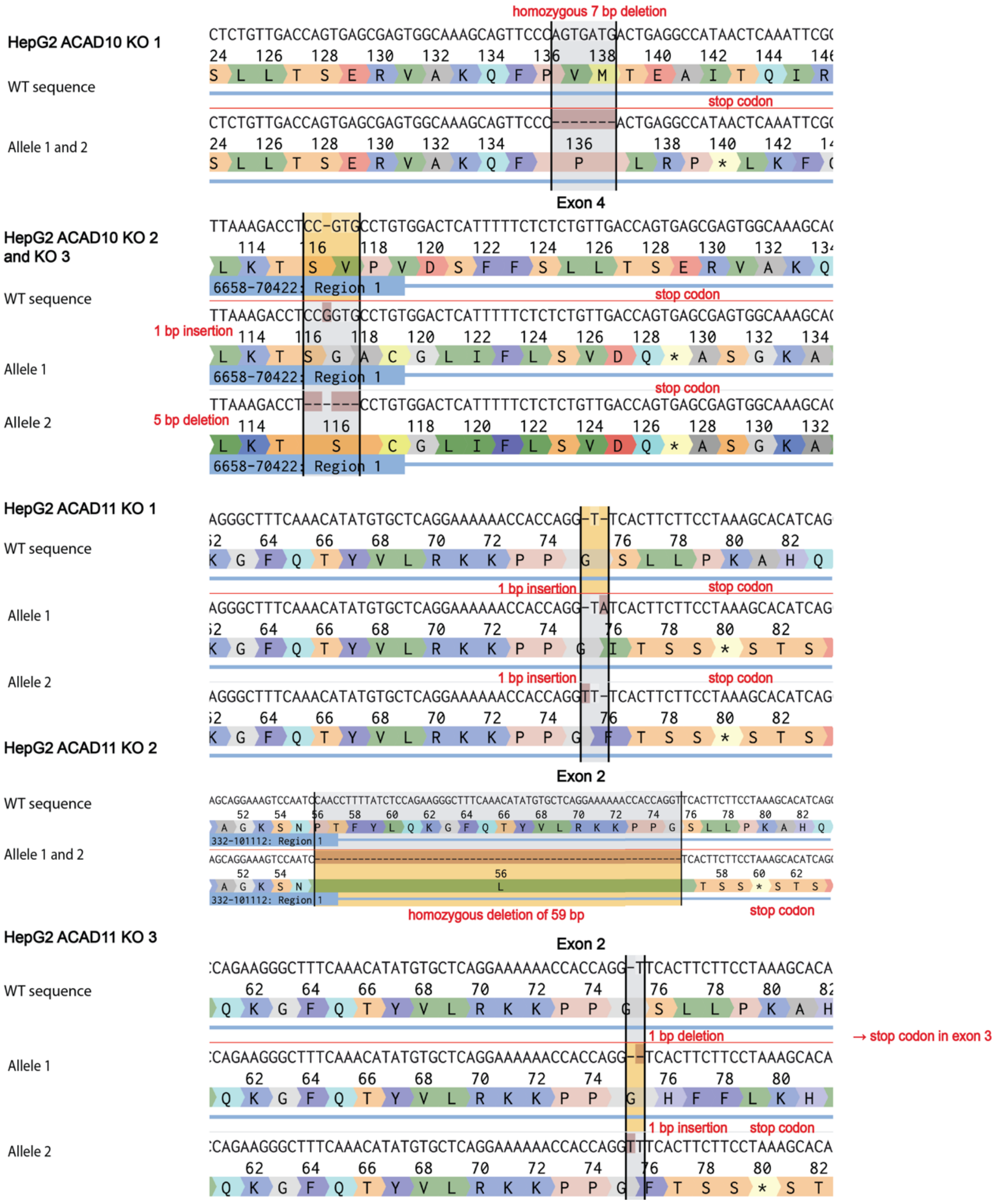

Fig. S3 Genotype of HepG2 knockout clones

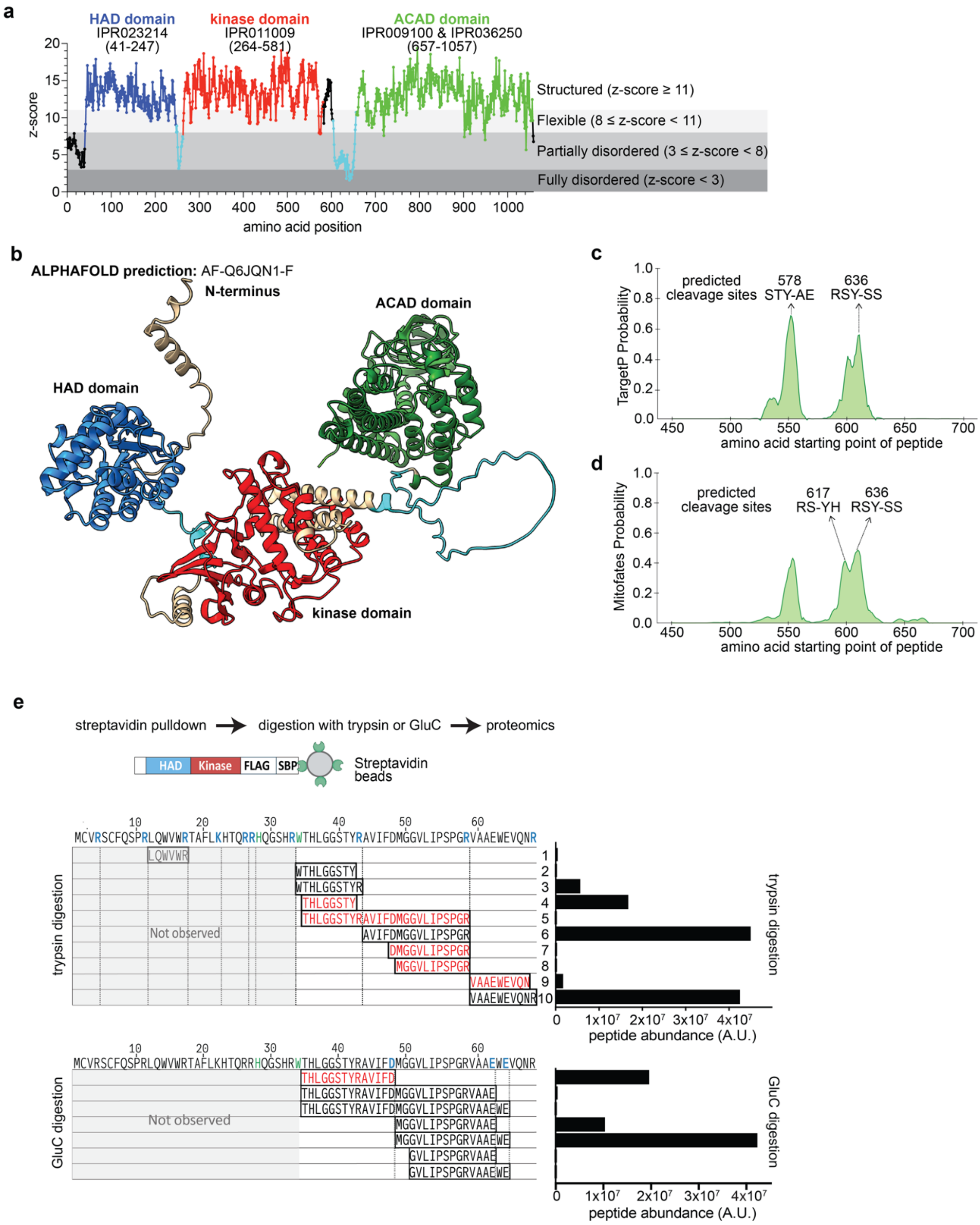

Fig. S4 Predicted structure and cleavage sites for human ACAD10
a, Prediction of unfolded regions within ACAD10 using the ADOPT (Attention DisOrder PredicTor) server [1]. **b,** Within the Alphafold predicted structure of human ACAD10 (AF-Q6JQN1-F1-model_v4), the HAD, the kinase and the ACAD domain are highlighted in blue, red and green respectively. The linker region is colored in cyan.
c-d, Prediction of cleavage sites by TargetP (**c**) and Mitofates (**d**). Successive one amino acid deletions of ACAD10 were submitted to the prediction algorithm.
e, Determination of the N-terminus of ACAD10. The first 608 amino acids of human ACAD10 were overexpressed with a C-terminal SFB tag in HEK293, and purified with streptavidin-Sepharose. A nano-LC-MS analysis after digestion with trypsin or GluC revealed that the mature protein starts with threonine 35. A.U. = arbitrary units. Peptides highlighted in red were generated by a cleavage independent of trypsin or GluC.

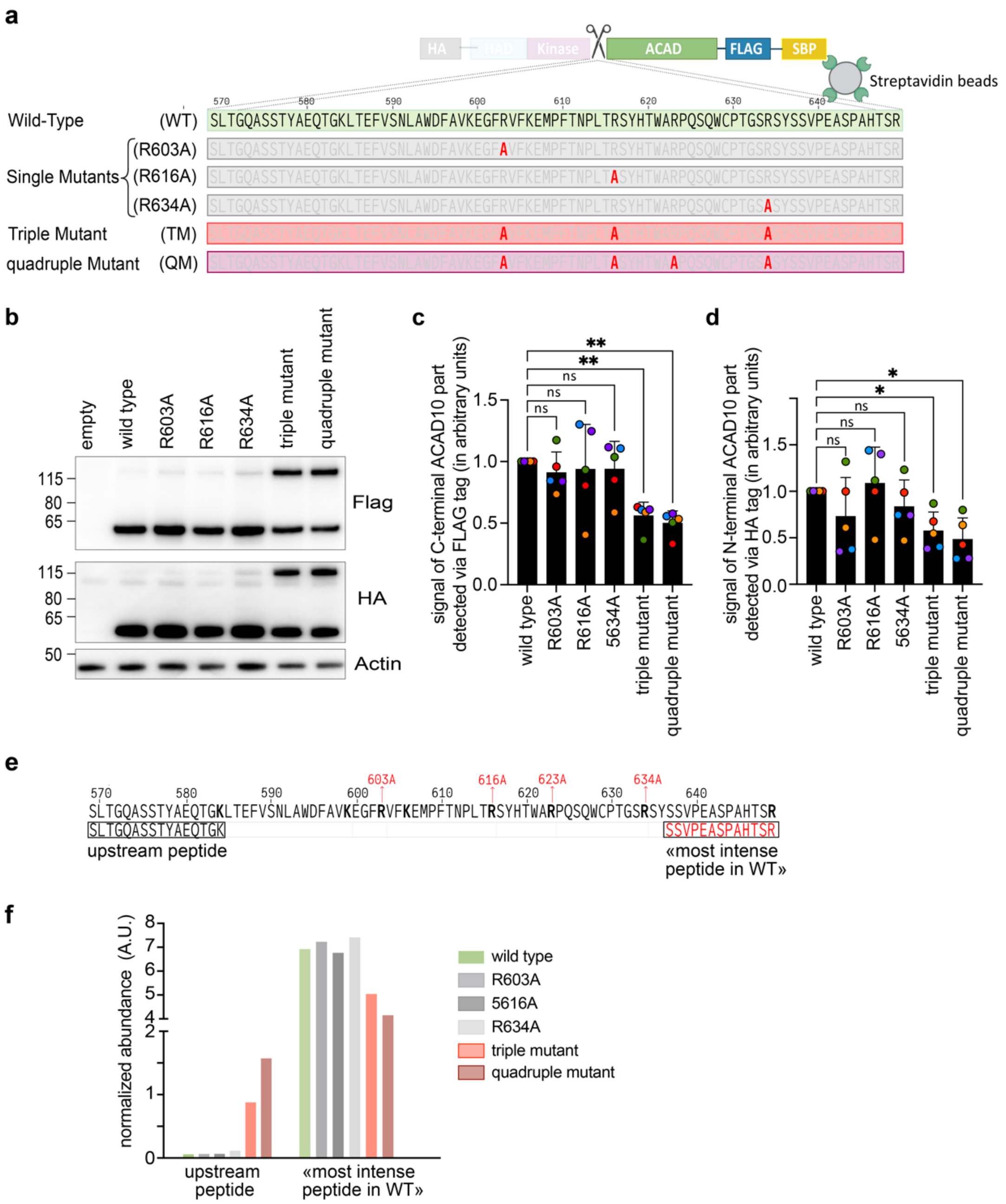

Fig. S5 Arginine residues within consensus MPP cleavage sites are required for optimal cleavage of ACAD10

a, Schematic representation of the mutations introduced into ACAD10. **b**, Western Blot analysis of ACAD10-deficient HepG2 cell lines upon re-expression of the indicated ACAD10 mutants. The anti-HA antibody recognizes the N-terminal part and the anti-FLAG antibody recognizes the C-terminal part of the protein. **c&d,** Quantification of the intensities of the C-terminal (FLAG-tag, panel c) and N-terminal (HA-tag, panel d) fragment of ACAD10 observed in western blot analyses from 5 independent experiments. Data are presented relative to the intensity of the wild type control. Asterisks indicate * $p<0.05$, ** $p<0.01$ in paired t-test after one-way ANOVA. **e&f,** Schematic representation of the analyzed tryptic peptides and quantification of the peptides obtained from the indicated proteins pulled down via the C-terminal SFB tag.

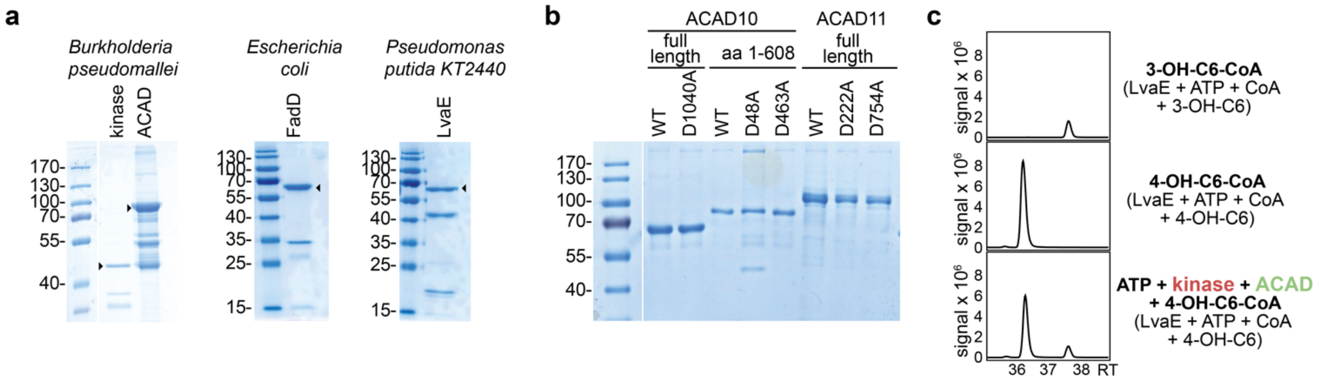

Fig. S6 Protein purification and metabolite standards

a, Purified bacterial proteins were analyzed by SDS-PAGE followed by Coomassie blue staining. This includes His-tagged *Burkholderia pseudomallei* kinase (42 kDa), MBP-tagged *Burkholderia pseudomallei* ACAD (86 kDa), His-tagged *E. coli* FadD (63 kDa), and His-tagged *Pseudomonas Putida* LvaE (60 kDa)

b, The indicated proteins were overexpressed as SFB-tagged fusion proteins (C-terminal for ACAD10 and N-terminal for ACAD11) followed by purification via streptavidin Sepharose. The following proteins were loaded (with the expected molecular weight in parentheses): Full length human ACAD10 WT (129.1 kDa) and mutant D1040A (129 kDa), as well as the N-terminal 608 amino acids of wild type, D48A mutant and D463A mutant ACAD10 (79 kDa). For ACAD11, the wild type protein as well as mutants D222A and D754A were purified (98 kDa).

c, Comparison of 4-OH-C6-CoA and 3-OH-C6-CoA in an extracted ion chromatogram for synthetic standards (upper two panels) and their presence in the indicated reaction of ACAD10 (lower panel).

	**P759 in ACAD10**<br>**P463 in ACAD11**<br>↓	**R913 in ACAD10**<br>**R627 in ACAD11**<br>↓
human ACAD10	CNCSAPDTGNMEL...	...EIAQGRLGPG
human ACAD11	FNCQAPDTGNMEV...	...EISQGRLGPG
Pseudomonas LvaC	FNCSAPDTGNMET...	...EIAQGRLGPG
Burkholderia LvaC	FNCNAPDTGNMET...	...EIAQGRLGPG
human MCAD	AI-E-GNSLGQMP...	...KVAMGAFDKT
human LCAD	GF-SIHSGIVMSY...	...YYIMKELPQE
human VLCAD	TL-GAHQSIGFKG...	...KVAMHILNNG
human ACAD9	TL-AAHQAIGLKG...	...KVAMNILNSG
human SCAD	IM-SVNNSLYLGP...	...KIAMQTLDMG

Fig. S7 Alignment of the sequence surrounding conserved proline and arginine residues in ACAD10 and ACAD11, their prokaryotic orthologs, and other ACADs.
Sequences were human ACAD10 (Q6JQN1), human ACAD11 (Q709F0), *Burkholderia pseudomallei* ACAD (Q63VL1), *Pseudomonas putida* ACAD (Q88K54), human MCAD (NP_000007.1), human LCAD (NP_001599.1), human VLCAD (NP_000009.1), human ACAD9 (Q9H845), and human SCAD (NP_000008.1). The alignment was generated with Clustal Omega [2].

Supplementary reference:

1. Redl I, Fisicaro C, Dutton O, Hoffmann F, Henderson L, Owens BMJ, Heberling M, Paci E, Tamiola K.
ADOPT: intrinsic protein disorder prediction through deep bidirectional transformers.
NAR Genom Bioinform. 2023;5(2):lqad041.
https://doi.org/10.1093/nargab/lqad041

2. Sievers F, Wilm A, Dineen D, Gibson TJ, Karplus K, Li W, Lopez R, McWilliam H, Remmert M, Soding J, Thompson JD, Higgins DG.
Fast, scalable generation of high-quality protein multiple sequence alignments using Clustal Omega.
Molecular systems biology. 2011;7:539.
https://doi.org/10.1038/msb.2011.75
